# Evaluating social protection mitigation effects on HIV/AIDS and Tuberculosis through a mathematical modelling study

**DOI:** 10.1038/s41598-024-62007-0

**Published:** 2024-05-23

**Authors:** Felipe Alves Rubio, Alan Alves Santana Amad, Temidayo James Aransiola, Robson Bruniera de Oliveira, Megan Naidoo, Erick Manuel Delgado Moya, Rodrigo Volmir Anderle, Alberto Pietro Sironi, José Alejandro Ordoñez, Mauro Niskier Sanchez, Juliane Fonseca de Oliveira, Luis Eugenio de Souza, Inês Dourado, James Macinko, Davide Rasella

**Affiliations:** 1 Institute of Collective Health (ISC), Federal University of Bahia (UFBA), Bahia, Brazil; 2 Center for Data and Knowledge Integration for Health, Salvador, Brazil; 3 ISGLOBAL, Hospital Clínic-Universitat de Barcelona, Barcelona, Spain; 4 Department of Public Health, University of Brasilia, Brasilia, Brazil; 5 College of Engineering, Swansea University, Bay Campus, Swansea, UK; 6 Department of Health Policy and Management, University of California, Los Angeles, USA

**Keywords:** Infectious diseases, Applied mathematics

## Abstract

The global economic downturn due to the COVID-19 pandemic, war in Ukraine, and worldwide inflation surge may have a profound impact on poverty-related infectious diseases, especially in low-and middle-income countries (LMICs). In this work, we developed mathematical models for HIV/AIDS and Tuberculosis (TB) in Brazil, one of the largest and most unequal LMICs, incorporating poverty rates and temporal dynamics to evaluate and forecast the impact of the increase in poverty due to the economic crisis, and estimate the mitigation effects of alternative poverty-reduction policies on the incidence and mortality from AIDS and TB up to 2030. Three main intervention scenarios were simulated—an economic crisis followed by the implementation of social protection policies with none, moderate, or strong coverage—evaluating the incidence and mortality from AIDS and TB. Without social protection policies to mitigate the impact of the economic crisis, the burden of HIV/AIDS and TB would be significantly larger over the next decade, being responsible in 2030 for an incidence 13% (95% CI 4–31%) and mortality 21% (95% CI 12–34%) higher for HIV/AIDS, and an incidence 16% (95% CI 10–25%) and mortality 22% (95% CI 15–31%) higher for TB, if compared with a scenario of moderate social protection. These differences would be significantly larger if compared with a scenario of strong social protection, resulting in more than 230,000 cases and 34,000 deaths from AIDS and TB averted over the next decade in Brazil. Using a comprehensive approach, that integrated economic forecasting with mathematical and epidemiological models, we were able to show the importance of implementing robust social protection policies to avert a significant increase in incidence and mortality from AIDS and TB during the current global economic downturn.

## Introduction

The COVID-19 pandemic has been one of the most impactful and complex events in recent human history. Over 120 million people have been pushed into extreme poverty, leading to the worldwide highest rise in social vulnerability since World War II^1^. Moreover, the global economic consequences of the recent war in Ukraine have pushed over 71 million more into severe poverty^2^. The worldwide inflationary crisis and looming global recession will further increase the number of individuals in extreme socioeconomic vulnerability^1–3^. Consequently, poverty-related diseases, which account for more than 45% of the disease burden in low- and middle-income countries (LMICs)^4^, will continue to spread among the more than 700 million people living in extreme poverty^2^. This will halt or reverse the progress made for most poverty-related infectious diseases, in particular Tuberculosis (TB) and HIV/AIDS, which have been responsible for more than 2.2 million deaths in 2021^5^. Moreover, this would endanger the achievement of the United Nation’s Sustainable Development Goal 3.3 to end the epidemics of AIDS and TB.

Poverty has consistently been recognized as one of the most important risk factors for TB^6^, and poverty-reduction programs have repeatedly shown a significant reduction in TB morbidity and mortality^7^. Statistical models have estimated that the elimination of extreme poverty would reduce the global incidence of TB by 33% by 2035^7^. While the evidence for HIV/AIDS is less consolidated, several studies have illustrated the impact of poverty-reduction policies on HIV prevention^8^, including the impact of conditional cash transfers in significantly reducing the HIV/AIDS burden^9,10^.

Brazil is among the LMIC with the highest number of Tuberculosis and HIV/AIDS cases, and one of the LMIC most severely impacted by the COVID-19 pandemic, with its second world’s highest deaths toll^11^. At the beginning of the COVID-19 pandemic, poverty rates rose all over the country^12^, but the prompt implementation of a large-scale Emergency Benefits Scheme (*Auxílio Emergencial*, AE) mitigated the impact of the crisis and reduced poverty to a historic low of five percent within a few weeks after AE implementation^13^. However, because the structural drivers of sustained poverty were still present, an abrupt pause in the AE program after a few months caused a relative increase of 329% in the poverty rate, reaching a historical high of 16% of the population in the first trimester of 2021^13^. Recent surveys report that food insecurity have affected 60% of the population, of which 15% live in hunger^14^.

The high number of TB and HIV/AIDS cases, the sharp increase in poverty rates during the pandemic, and the implementation of large-scale social protection policies such as the AE, make Brazil a unique country to assess the impact of economic crises on these two infectious diseases. In this study, we aimed to evaluate and forecast the impact of the increase in poverty from the economic crisis, and estimate the mitigation effects of alternative poverty-reduction policies on the incidence and mortality from AIDS and TB.

## Methods

### Epidemiological transmission models

**Figure 1:**
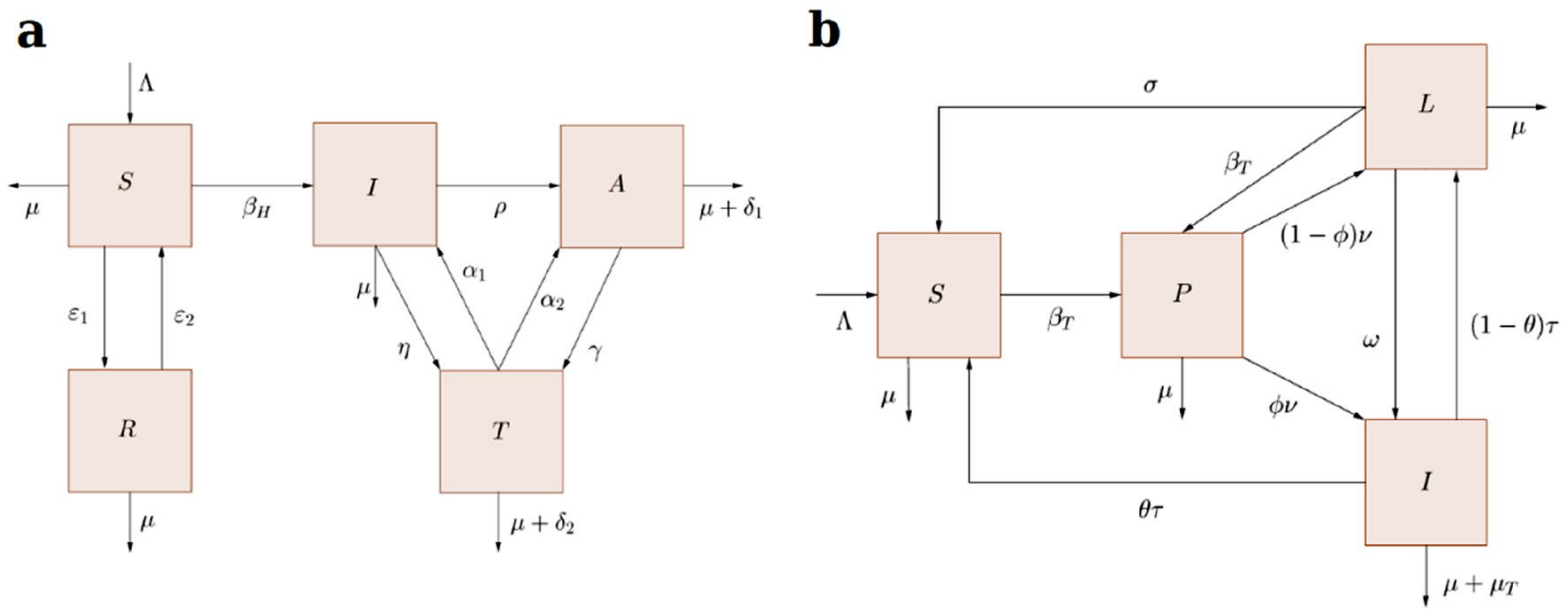
Structure of the compartmental models for HIV/AIDS and Tuberculosis. (**a**) HIV/AIDS transmission model: (S) those susceptible to HIV infection via sexual contact, (I) HIV-positive individuals who are infectious, (A) individuals with full-blown AIDS, (T) individuals being treated and who therefore have an undetectable viral load, (R) individuals who are not yet sexually active or have changed their sexual habits, so they are no longer part of the susceptible population. (**b**) Tuberculosis transmission model: (S) susceptible, (P) primary infection, (L) latent infection, (I) active tuberculosis disease.

The model for HIV/AIDS transmission dynamics was based on previous studies^15^ and was composed of nonlinear ordinary differentiation equations and five dynamic variables: those susceptible to HIV infection via sexual contact ($S$), HIV-positive individuals who are infectious ($I$), individuals with full-blown AIDS ($A$), individuals being treated and who therefore have an undetectable viral load ($T$), and individuals who are not yet sexually active or have changed their sexual habits, so they are no longer part of the susceptible population ($R$). The TB model, based on previous studies^16^, was composed of nonlinear ordinary differential equations and four dynamic variables: susceptible ($S$), primary infection ($P$), latent infection ($L$), and active tuberculosis disease ($I$). Figure 1 presents the structure of the two compartmental models (for a full description of the models and system of equations see Supplementary Material pages 2–5). The comorbidity of HIV/AIDS and TB was not included in the models because it represents less than 10% of TB cases in Brazil, and therefore would not significantly modify the dynamics of the two diseases^17,18^.

### Incorporating poverty into mathematical modelling

Several factors, from socioeconomic improvements in society to healthcare advancements (e.g., new diagnostics, therapeutics, or new protocols), can significantly influence the dynamics of HIV/AIDS and TB. However, the influential drivers that rapidly change and experience large variations during an economic crisis are poverty rates and socioeconomic vulnerabilities^2,7,19^.

In accordance with theoretical and empirical considerations in including social determinants of health (SDH) in infectious diseases model^20^, we incorporated the annual poverty rates in all model parameters sensitive to poverty and social vulnerabilities in HIV/AIDS and TB according to the literature (see Supplementary Material pages 5–6)^7,9,21^.

Moreover, in order to take into account changes in other socioeconomic conditions and advances in healthcare, which have been improving over the last two decades in the country^17,18,22^, we also included a decreasing time trend in the model, for that a time-dependent term was included in the beta parameter (see Supplementary Material pages 6–7). We have chosen the beta parameters because the most significant societal changes in Brazil in the last two decades, and potentially up to 2030, have been the reduction of poverty and socioeconomic inequalities, and the improvement of social determinants of health. These determinants are highly influential and are mainly acting on the reduction of the risk of becoming infected (and consequently in our models in the Beta parameters) through several mechanisms: more related to sexual behaviors for HIV/AIDS^23,24^, and to nutritional status and housing conditions for TB^25,26^. Moreover, the most promising biomedical interventions that will be implemented or scaled up in the next decade to reduce HIV/AIDS and TB burden are focused on preventing the infection. One of the most studied interventions at the moment is the use of PrEP (pre-exposure prophylaxis): when taken as prescribed, PrEP is highly effective in preventing HIV infections^27^. Regarding TB, the anti-tuberculosis drugs for the treatment of latent tuberculosis infection^28^ and the tuberculosis vaccine will also reduce active TB infections^29^.

Consequently, in order to keep a simpler model structure, we assumed that other potential factors (such as the introduction of new treatments) would not meaningfully influence the dynamics of the two diseases and consequently their projections up to 2030. As a matter of fact, from the sensitivity analysis, the beta (transmission parameter) was the most influential parameter for both HIV/AIDS and TB, while the other parameters were less affecting the future trends in disease incidence and mortality. Moreover, the aim of our study was not to specifically predict the AIDS and TB burden during the next decade, but to estimate the difference between AIDS and TB projections according to alternative policy responses. All factors and historical events not included in our models would have similarly affected all the scenarios under comparison^30,31^, and would not have significantly changed the magnitude of their comparison estimates.

### Estimating poverty trends and the effects of mitigation strategies on poverty

The calculation of poverty rates for the period 2000–2019 is described in details in the Supplementary (see Supplementary Material page 13–16). Regarding the social protection policies that could be implemented to mitigate the impact of the economic crisis, we focused on poverty-reduction policies for two reasons: first, these policies, particularly cash transfers (CT) programs, are among the social protection policies that can be implemented relatively quickly, widely, and effectively during a crisis^2^. Second, poverty rates have been consistently responsive to these policies, as shown by the Bolsa Familia conditional CT program in the last two decades and by the AE CT during the pandemic^9,13^. While poverty-reduction policies may also affect other socioeconomic indicators, such as educational-level and household infrastructure, they likely will have a smaller and delayed effect on HIV/AIDS and TB in the middle- and long- term.

In this study, several scenarios of poverty rates were forecast until 2030, based on potential developments in the economic crisis and implementation of alternative social protection policies (for full description see Supplementary Material pages 16–17). The main analyses showed the three most relevant scenarios: strong social protection, moderate social protection, and no social protection. The objective was to evaluate how differences in poverty-levels from varying social protection policies would affect incidence and mortality from AIDS and TB, focusing on comparisons between scenarios more than on predictions of poverty rates during the study period.

The first scenario of strong coverage assumed that an AE-like intervention, based on cash transfers, is implemented and sustained even after the pandemic, keeping poverty at extremely low rates until 2030. The scenario was developed from National Household Surveys (PNAD) microdata up to 2020, the year in which the AE was implemented during the pandemic, followed by an extrapolation of poverty rates up to 2030 using an exponential decay function previously calibrated with real data, as done in previous studies^7,31^. The second scenario assumed a moderate-level of social protection similar to pre-pandemic years. Here, the AE social protection is implemented only in 2020 followed by the maintenance of existing social protection policies. In this scenario, the poverty rate estimates from the year 2001 to 2020 were used to forecast values from 2021 to 2030 using Vector Autoregression (VAR) models (see Supplementary Material page 13). The third scenario of no coverage was based on data from PNAD up to 2019. The poverty rates of 2020 were obtained by subtracting the AE money allowances (available in the survey microdata) from the income per capita of the AE beneficiaries in the PNAD 2020 data, thereby estimating the poverty rates in a pandemic scenario where AE was not implemented. The trends in poverty were forecast up to 2030 through extrapolation using an exponential function similar to the one used for the strong social protection scenario, calibrated using 2019–2020 poverty rates estimated for this scenario, as done in previous studies^31^. It was assumed a gradual decrease in poverty rates starting from the middle of the decade (2026), estimated according to the behavior of previous economic recessions in the country (other lengths of the crisis have been tested as sensitivity analyses)^31^.

### Estimation of the parameters

The birth and natural mortality rates were estimated using counts and projections of the Brazilian population between 2003 and 2030. We calibrated the parameters of the AIDS model using reported yearly data on newly notified AIDS yearly cases over the period 2003–2019 (Supplementary Material pages 11–12)^18^. We did not use data for the year 2020 to avoid the underreporting issues due to the pandemic, which could affect the calibration process. We used a genetic algorithm (GA) to fit the HIV/AIDS and TB models to the notified yearly AIDS cases and AIDS-related death and reported yearly TB cases and TB-related death data, respectively (see Supplementary Material pages 8–9). The method consisted of fitting both new yearly cases and deaths at the same time. As an inverse problem, we obtained the parameters of the model that resulted in the smallest error using the RMSE formula.

Additionally, to address potential uncertainty in the AIDS and TB administrative data and therefore the calibrated parameters of the models, we estimated prediction intervals for each AIDS and TB projected scenario. We used a non-parametric bootstrapping method with a Poisson distribution, developing 100 replicates of the original data during the period 2003–2019 for AIDS and TB incidence and deaths, as done in previous modelling studies in Brazil^32^. We successively estimated the parameters of the models, with the GA methods described above, for each one of the 100 replicates. To generate the uncertainty intervals, we used the bootstrapping approach described above, which consisted of creating a 100 sample of new cases and deaths (for each disease separately) and applying the calibration method for each of them. Once we had a deterministic system from the calibration method we obtained a time series (output) for each element (time series of new yearly cases and deaths) of the calibrated sample. With the 100 sets of parameters, we estimated 100 trajectories of AIDS and TB incidence and deaths for each one of the poverty-related scenarios described above (Supplementary Material pages 10–12). We calculated the incidence and mortality rates of AIDS and TB by dividing the number of cases and deaths for each disease per 100,000 person-years. Therefore, because of the bootstrapping process, when calculating the 95% UI we could have this tight model fit and some data points outside the uncertainty range. However, even with this limitation we can see the trends are respected in both models.

## Results

**Figure 2:**
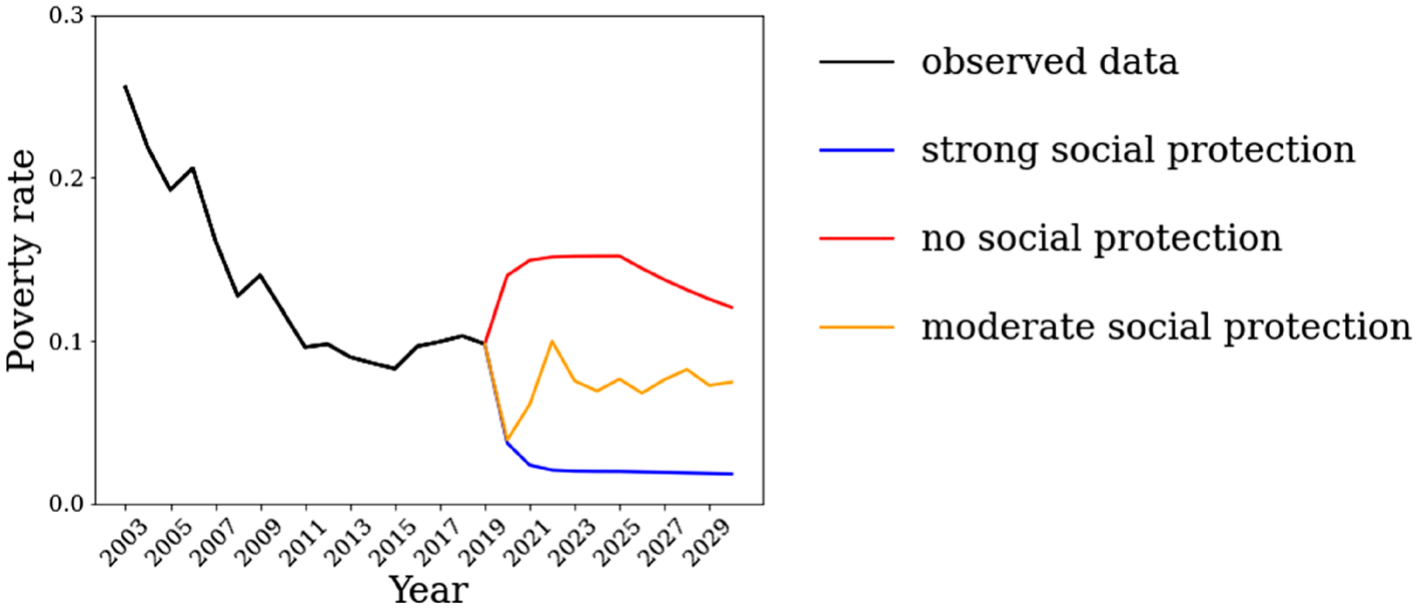
Time series of the poverty rates in Brazil between 2003 and 2019, and potential scenarios between 2020 and 2030. Poverty Rates are expressed as proportion (from 0 to 1).

Figure 2 shows the poverty rates using the real data from 2001 to 2020, and the three simulated scenarios from 2021 to 2030. There was a steady reduction in poverty from the beginning of 2001 until the Brazilian economic recession of 2014, when poverty started to increase until 2020, the first year of the pandemic. The scenario of no social protection (Fig. 2 red line) illustrates how the lack of state intervention during the pandemic could cause a substantial increase in poverty. This would only start to decrease after the potential end of the economic crisis, reaching 12.01% in 2030. The strong social protection scenario (Fig. 2 blue line) portrays the situation whereby the AE social protection policy is implemented and sustained even after the pandemic, causing the reduction and stabilization of poverty rates, reaching 1.81% in 2030. The moderate social protection scenario (Fig. 2 orange line) is where strong the AE social protection is implemented only in 2020 and, thereafter, followed by the maintenance of existing social protection policies prior the pandemic in the subsequent years; hence, it continues the slight trend of poverty decrease from the previous decade, reaching 7.45% in 2030.

**Figure 3:**
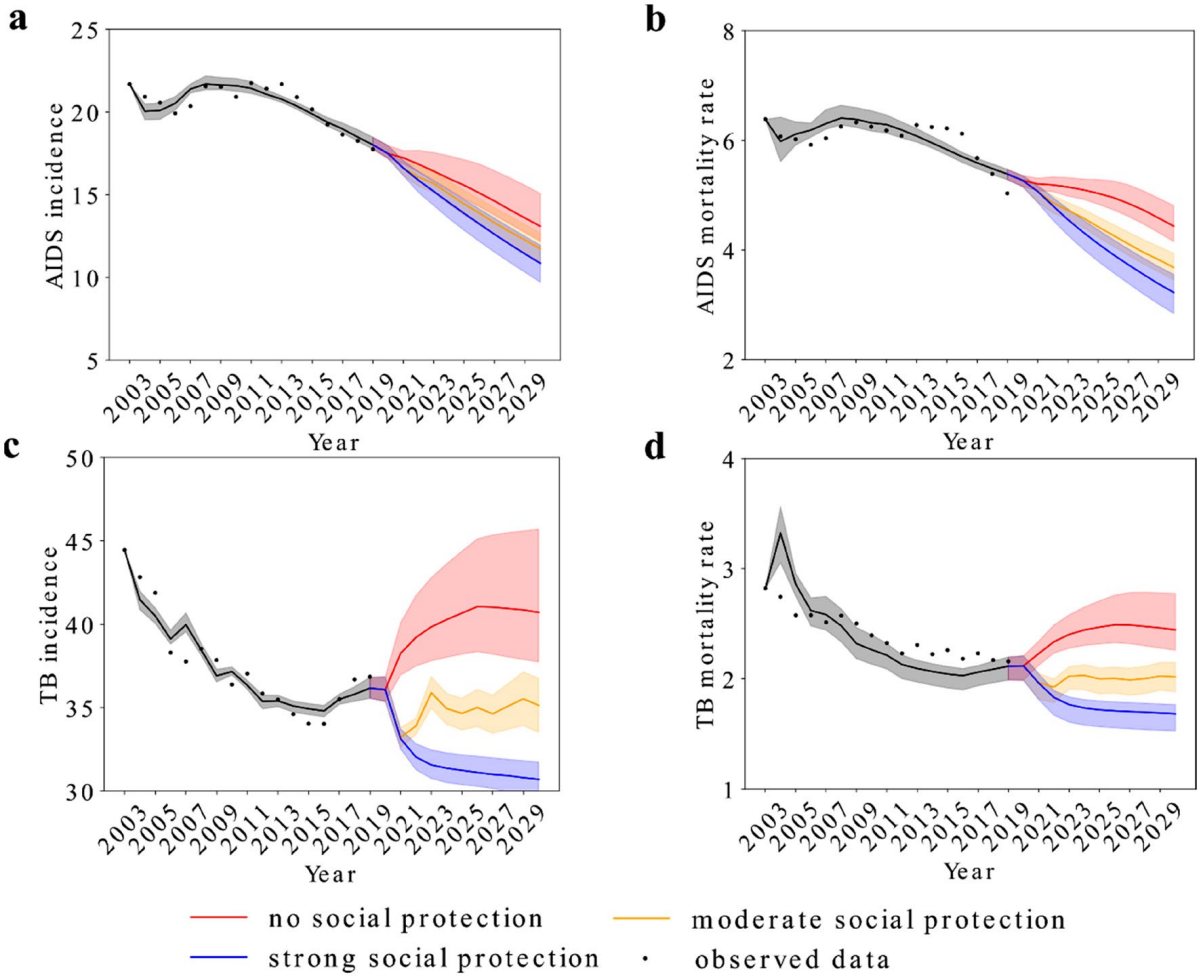
Incidence and mortality rates due to AIDS and Tuberculosis from 2003 to 2030 according to different social protection scenarios. (**a**) AIDS incidence under different scenarios of social protection between 2003 and 2030, (**b**) AIDS-related mortality under different scenarios of social protection between 2003 and 2030, (**c**) Tuberculosis incidence under different scenarios of social protection between 2003 and 2030, and (**d**) Tuberculosis mortality under different scenarios of social protection between 2003 and 2030. Shaded bands represent 95% of simulated predictions by social protection level.

Figure 3 shows that the incidence and mortality from AIDS and TB will follow different trends depending on the poverty rate and level of social protection. AIDS incidence and AIDS-related mortality have been on a downward trend in the previous decade. But with no social protection, the sudden increase in poverty rates due to the economic crisis and the lack of social protection policies will hamper this trend, while a strong social protection response would accelerate it, notably for AIDS-related mortality. A moderate social response will continue the incidence and mortality trends of the previous decade.

Regarding TB, with no social protection, incidence and mortality rates will increase considerably, with a tendency to slightly decrease in the years closer to 2030. In a strong social protection scenario, both incidence and mortality will notably decrease in the first years of the current decade, and will start of slightly decreasing in the middle of the forecast period, while in a moderate social protection scenario incidence and mortality will essentially stabilize, following the trend of the previous decade. Overall, the difference between scenarios was substantially larger for TB than for AIDS.

Tables 1 and 2 show the Rate Ratio (RR) for incidence and mortality in different years of the study period, using the moderate social protection scenario as reference, and comparing it with strong or no social protection scenarios. The moderate social protection scenario was considered the most plausible if social protection policies were to be preserved in the country over the next years.

**Table 1:** AIDS incidence and mortality rate ratios and avoidable cases and deaths.

Year	No social protection		Strong social protection	
	Rate ratio	95% CI	Rate ratio	95% CI
	Incidence			
2021	1.04	[1.02; 1.09]	0.9994	[0.998; 0.9997]
2025	1.08	[1.02; 1.20]	0.96	[0.91; 0.99]
2030	1.13	[1.04; 1.31]	0.92	[0.85; 0.97]
Avoidable cases (2020–2030)	25,548	[7858, 59,205]	− 12,656	[4507; 25,561]

	Mortality			
2021	1.03	[1.02; 1.07]	0.9995	[0.998; 0.9997]
2025	1.14	[1.08; 1.22]	0.93	[0.89; 0.96]
2030	1.21	[1.12; 1.34]	0.87	[0.82; 0.92]
Avoidable deaths (2020–2030)	12,887	[7789; 19,093]	− 6480	[4240; 9486]

AIDS incidence and mortality rate ratios for specific years with 95% confidence intervals (CI). Avoidable AIDS cases and deaths from 2020 to 2030 with 95% CI. Moderate social protection scenario as the reference category.

**Table 2:** Tuberculosis incidence and mortality rate ratios and avoidable cases and deaths.

Year	No social protection		Strong social protection	
	Rate ratio	95% CI	Rate ratio	95% CI
	Incidence			
2021	1.15	[1.11; 1.22]	0.997	[0.996; 0.998]
2025	1.17	[1.12; 1.25]	0.90	[0.87; 0.94]
2030	1.16	[1.10; 1.25]	0.88	[0.83; 0.92]
Avoidable cases (2020–2030)	120,960	[79,679; 182,898]	− 74,429	[51,224; 108,495]

	Mortality			
2021	1.14	[1.11; 1.20]	0.997	[0.996; 0.998]
2025	1.25	[1.18; 1.34]	0.85	[0.82; 0.89]
2030	1.22	[1.15; 1.31]	0.82	[0.78; 0.87]
Avoidable deaths (2020–2030)	9781	[7229; 13,382]	− 5627	[4331; 7412]

Tuberculosis incidence and mortality rate ratios for specific years with 95% confidence intervals (CI). Avoidable Tuberculosis cases and deaths from 2020 to 2030 with 95% CI. Moderate social protection scenario as the reference category.

The highest RR for AIDS was in 2030, corresponding to 13% (95% CI 4–31%) and 21% (95% CI 12–34%) higher incidence and mortality, respectively, in the case of no social protection. In case of strong social protection, there would be lower incidence and mortality at 8% (95% CI 3–15%) and 13% (95% CI 8–18%), respectively. Regarding TB, the highest RR corresponded to 17% (95% CI 12–25%) and 25% (95% CI 18–34%) higher incidence and mortality rates, respectively, in 2025 if no social protection was implemented. In the case of strong social protection, incidence and mortality rates would be 12% (95% CI 8–17%) and 18% (95% CI 13–22%) lower in 2030.

In terms of avoidable cases and deaths, if no social protection policies will be implemented during the acute phase of the recession and afterward, and comparing with a moderate social protection, the number of avoidable new cases of TB over the period 2020–2030 will be nearly 5 times higher than that for AIDS, reaching 120,960 for TB (95% CI 79,679–182,898) versus the 25,548 (95% CI 7,858–59,205) for AIDS. However, the number of averted deaths will be higher for AIDS (12,887; 95% CI 7789–19,093) than for TB (9781; 95% CI 7,229–13,382) from 2020 to 2030. These differences would be significantly larger if the scenario of no social protection is compared with a scenario of strong social protection, resulting in more than 230,000 cases and 34,000 deaths of AIDS and Tuberculosis that could be averted over the next decade if the second policy option was implemented.

Several alternative economic scenarios, developed as sensitivity analyses (see Supplementary Material page 16), showed estimates compatible and convergent—in terms of direction and magnitude—with the findings described above.

## Discussion

Our results show that the consistent growth of poverty rates due to current economic crisis could significantly increase AIDS and TB morbidity and mortality over the next decade, causing thousands of avoidable cases and deaths, while social protection policies could significantly mitigate this impact.

Previous studies have shown the mitigating effects of poverty-reduction policies on the increase of overall mortality during economic recessions^31,33^, but the impact of such policies on poverty-related infectious diseases has never been evaluated. A recent study evaluated retrospectively the impact of the past 2015–2016 Brazilian economic crisis on the increase of TB incidence^34^: interestingly, while the growth of poverty rates was lower than the one we modelled in our study for the current crisis, the magnitude of the estimated relationship between the increase in poverty rates and the corresponding increase in TB incidence was similar, despite the use of different modelling techniques.

While mathematical models of the transmission dynamics for HIV/AIDS and TB are widely used in the literature to evaluate the impact of risk factors or control interventions^15,16^, the inclusion of SDH in these models is still in its infancy^20^. Changes in SDH, and in particular in poverty and levels of socioeconomic vulnerability, could strongly affect the dynamics of these infectious diseases^20^. Complete descriptions and theoretical frameworks of the effects of poverty, and other SDH, on all the steps of the disease progression in HIV/AIDS and TB is provided in the Supplementary Material pages 27–29.

Our study has limitations. First, the inclusion of poverty as the sole indicator of deprivation and social vulnerability. While this was necessary to avoid overfitting—also considering the limited number of real data, we assumed that poverty rates acted as proxy of a wider range of socioeconomic vulnerabilities associated with HIV/AIDS and TB, determining -from upstream- more proximal risk factors. For this reason, poverty rate was included in all relevant parameters of both compartmental models. Furthermore, a time factor was also incorporated and calibrated, representing other improvements in socioeconomic conditions and advancements in healthcare. Second, while we did not include other factors (such as the potential introduction of new treatments or diagnostics) that could influence the dynamics of the two diseases and consequently their projections, the aim of our study was not to specifically predict the AIDS and TB burden during the next decade, which would have required strong assumptions and high uncertainty. The goal of our study was to estimate the difference between AIDS and TB projections according to potential evolutions of the economic crisis and alternative policy responses. In this sense, we assumed that all factors and historical events not included in our models would have affected the scenarios and disease trends similarly, and would not have significantly changed the magnitude of their comparisons. Third, although already used in similar studies^15,16^, our compartmental models for HIV/AIDS and TB do not consider more complex disease status, such as differences in treatment compliance, TB resistance, and the multi-morbidity of HIV/AIDS and TB. While this was necessary to incorporate poverty and time factors into the models, it is also unlikely to have compromised the resulting trends, considering Brazil’s treatment compliance for TB and HIV/AIDS is relatively high in comparison to other LMICs, TB resistance is below 1%, and HIV/AIDS-TB multi-morbidity is less than 10%^17,18^.

Finally, while poverty trends and exponential decay curves were based on previously published studies and calibrated on real data^7,31^, they model the future behavior of the economic crisis based on theoretical considerations. For these reasons, several alternative economic scenarios were simulated (Supplementary Material pages 16–17), showing comparable estimates with the ones presented in the main results. In summary, while the models used for our study have a low level of complexity in terms of the number of compartments and their relationships, this was required by the inclusion and calibration of the poverty terms in many of its parameters. The use of more complex compartmental models, while would have changed some temporal dynamics in the response of incidence and mortality rates to poverty fluctuations, should not have meaningfully changed our finding in the middle and long term.

## Conclusion

To our knowledge, this is one of the first modelling study to incorporate poverty and vulnerability measures into a HIV/AIDS and TB mathematical models allowing to evaluate the impact of an economic crisis on poverty-related infectious diseases. Our models predict that the implementation of robust social protection policies could avert significant increases in the incidence and mortality from AIDS and TB, and that the lack of policy responses during economic crises could be responsible for a significantly higher burden of HIV/AIDS and TB, potentially hampering the achievement of Sustainable Development Goal 3.3.

### Supplementary Information

Supplementary Information.

## Data Availability

The AIDS and TB datasets generated and/or analysed during the current study are available in the Brazilian Ministry of Health repository, http://antigo.aids.gov.br/pt-br/pub/2020/boletim-epidemiologico-hivaids-2020 and http://antigo.aids.gov.br/pt-br/pub/2020/boletim-epidemiologico-de-turbeculose-2020, respectively.
