# Supplementary material for: Evaluating social protection mitigation effects on HIV/AIDS and Tuberculosis through a mathematical modelling study
Source: Sci Rep. 2024 May 23;14:11739. doi: 10.1038/s41598-024-62007-0 (PMC11111786; doi:10.1038/s41598-024-62007-0)
Supplement: Supplementary file 1 — Supplementary Information. [file 41598_2024_62007_MOESM1_ESM.pdf]

Evaluating social protection mitigation effects on HIV/AIDS and Tuberculosis through a mathematical modelling study

Rubio et. al.

HIV/AIDS transmission model

We based the mathematical model for HIV/AIDS transmission dynamics on the work of Huo et al. (2016). The population is divided into five compartments: susceptible to HIV infection via sexual contact (S), HIV-positive individuals who are infectious (I), individuals with full-blown AIDS (A), individuals being treated with undetectable viral load (T), and individuals who are not yet sexually active or have changed their sexual habits so they are no longer part of the susceptible population (R)[1].

Susceptible individuals have a constant per-capita birth rate Λ and a per-capita death rate μ. These individuals can be infected through contact with HIV-positive individuals (effective contact rate β_H). Some susceptible individuals change their sexual habits at a per-capita rate, ε_1, removing them from the susceptible population. However, some individuals in the R class can neglect their safe sexual habits and return to the susceptible compartment at a per-capita rate, ε_2. HIV-positive individuals after a period ρ^{-1} become individuals with full-blown AIDS as a result of not receiving and/or adhering to treatment. Conversely, HIV-positive individuals receiving and adhering to treatment after a period η^{-1} reach an undetectable viral load. If the treatment is stopped, the individuals in the T class can increase their viral load and become infectious (I) or even have full-blown AIDS (A) at a per-capita rate α_1 and α_2, respectively. We also consider full-blown individuals receiving treatment can move to compartment T at a per-capita rate γ. Individuals in the T or A classes have a disease-related death rate of δ_1 and δ_2, respectively. The mathematical model is given by the following nonlinear system of ordinary differential equations:

$$\frac{dS}{dt}=\Lambda-\beta_H\frac{IS}{N}-(\varepsilon_1+\mu)S+\varepsilon_2 R$$

$$\frac{dI}{dt}=\beta_H\frac{IS}{N}+\alpha_1 T-(\mu+\rho+\eta)I$$

$$\frac{dA}{dt}=\rho I+\alpha_2 T-(\mu+\delta_1+\gamma)A \quad (1)$$

$$\frac{dT}{dt}=\eta I+\gamma A-(\alpha_1+\mu+\delta_2+\alpha_2)T$$

$$\frac{dR}{dt}=\varepsilon_1 S-(\varepsilon_2+\mu)R$$

with $S(0)>0, I(0)\geq 0, A(0)\geq 0, T(0)\geq 0, R(0)\geq 0$, and N represents the total population, which is given by $N=S+I+A+T+R$. Table S1 describes the nonnegative parameters of the system (1). Figure 1a in the main text shows the epidemiological scheme.

Parameter	Description	Value	Reference
Λ	Birth rate	4,590,490.56	Derived from [2]
μ	Natural mortality rate	1/75.50	Derived from [2]
ε_1	Rate of susceptible individuals who changed their habits	0.03	Derived from [1]
ε_2	Rate of susceptible individuals who changed their habits and return to the susceptible compartment	0.003	Derived from [1]
β_H	HIV transmission rate	(0,10)	Assumed
ρ	Progression rate to A from I	(1/15,1/10)	[3]
η	Progression rate to T from I	2	[4]
α_1	Treatment failure rate (T to I compartmental)	(0,0.4)	[5–8]
α_2	Treatment failure rate (T to A compartmental)	(0,0.4)	[5–8]
δ_1	AIDS-related death rate (individuals with full-blown Aids)	(0,0.1)	[9]
δ_2	AIDS-related death rate for individuals being treated	0.0667	[9]
γ	Progression rate to T from A	(0.2,2)	[4,10]

Table S1: Parameter description of the HIV/AIDS model

Tuberculosis transmission model

The tuberculosis model was based on the work of Gomes et al. (2019), which presented a mathematical model composed of four nonlinear ordinary differential equations[11], which represents susceptible (S), primary infection (P), latent infection (L), and active tuberculosis disease (I). We assume that only susceptible individuals have a birth rate Λ but all compartments have a natural death rate μ. Contact with an infectious individual (I), represented by the parameter β_T, results in a susceptible individual becoming infected with a primary infection (P). After a period ν^{-1} individuals in the compartment P, a fraction ϕ acquire active tuberculosis, while a fraction $(1-\phi)$ move to the latent compartment (L). Individuals with active tuberculosis have an additional mortality rate μ_T due to the tuberculosis disease. We consider that through treatment, represented by the per-capita rate τ, a fraction θ completely eliminate the bacteria, which makes this individual return to the susceptible compartment. On the other hand, a fraction $(1-\theta)$ cannot eliminate the bacteria and the infection becomes latent (L). These individuals in the latent compartment can become infectious when in contact with infectious individuals I or through reactivation and will return to the compartment I at a per-capita rate ω. We also assume that latent individuals can return to the susceptible compartment at a per-capita rate σ. The system of equations is given explicitly below:

$$\frac{dS}{dt}=\Lambda-\mu S-\beta_T\frac{IS}{N}+\theta\tau I+\sigma L$$

$$\frac{dP}{dt}=\beta_T\frac{(IS+LI)}{N}-(\nu+\mu)P \quad (2)$$

$$\frac{dI}{dt}=\phi\nu P+\omega L-(\mu_T+\mu+\tau)I$$

$$\frac{dL}{dt}=(1-\theta)\tau I+(1-\phi)\nu P-\beta_T\frac{LI}{N}-(\omega+\mu+\sigma)L$$

with nonnegative initial conditions and N is the total population defined by $N = S + P + I + L$. The nonnegative parameters of the system (2) are described in Table S2. Figure 1b in the main text presents the schematic diagram.

Parameter	Description	Value	Reference
Λ	Birth rate	4,590,490.56	Derived from [2]
μ	Natural mortality rate	1/75.50	Derived from [2]
β_T	TB transmission rate	(0,10)	Assumed
θ	Proportion clearing infection upon treatment	0.75	[12,13]
ν	Rate of progression from primary infection	(2,4)	[11]
ϕ	Proportion progressing from primary infection to active disease	0.05	[11,14–16]
τ	Rate of successful treatment	(1/1.5,2)	[11–13]
ω	Rate of reactivation of latent infection	(0.001, 0.1)	[11,14–16]
μ_T	TB-related death rate	(0.03, 0.08)	[17]
σ	Progression rate to S from L	0.01	Assumed

Table S2: Parameter description of the Tuberculosis model

Incorporating poverty into mathematical modelling

For the HIV/AIDS model, we selected the parameters influenced by the poverty rate are

$$\beta_H(t) = \beta_{H1} + \beta_{H2} p(t)$$

$$\delta_1(t) = \delta_{11} + \delta_{12} p(t)$$

$$\alpha_1(t) = \alpha_{11} + \alpha_{12} p(t) \quad (3)$$

$$\alpha_2(t) = \alpha_{21} + \alpha_{22} p(t)$$

$$\rho(t) = \rho_1 + \rho_2 p(t)$$

$$\gamma(t) = \gamma_1 - \gamma_2 p(t)$$

where $0<p(t)<1$ represents the proportion of people living in poverty in year t.

For the TB model (2), we assumed that the following parameters are influenced by the poverty rate $p(t)$:

$$\beta_T(t)=\beta_{T1}+\beta_{T2}p(t)$$

$$v(t)=v_1+v_2p(t)$$

$$\omega(t)=\omega_1+\omega_2p(t)(4)$$

$$\mu_T(t)=\mu_{T1}+\mu_{T2}p(t)$$

$$\tau(t)=\tau_1-\tau_2p(t)$$

Therefore, to incorporate the poverty rate, we included the parameter formulation given by (3) and (4) in the models (1) and (2), respectively. Specifically, for HIV/AIDS: the effective contact rate (and consequently the probability of being infected), the progression from HIV to AIDS, the treatment adherence and outcomes, and the case-fatality rate. For TB: the effective contact rate, the progression from latent TB infection to active TB disease, the treatment adherence and outcomes, including cure rate, and the case-fatality rate. The strength of the poverty rate indicator in each one of the parameters above, and in the overall disease dynamics, was determined through the calibration of the model with real world data. Poverty is one of the most consolidated determinants of Tuberculosis and HIV/AIDS,[29,31,34] especially in LMIC, with a well-established causal relationship, and its introduction in the calibration process allow to estimate its influence within the infectious diseases dynamics in the Brazilian context. This was possible because poverty – as proxy of socioeconomic vulnerabilities - has been the only factor during the last two decades to present an alternance of decreasing and increasing -in correspondence of economic crises- trends. However, only

considering poverty in the parameters is overly simplistic and can overestimate the effect of poverty on incidence and mortality. This is because there will likely be improvements in surveillance, drugs, and other advancements that will contribute to the reduction of transmission. Therefore, we added a reduction term $(-\beta_{i3}t)$ in the transmission rates (β_H and β_T) to represent this effect in each model. Here, we assume $\left(k_1\overline{\beta_P}/T_Y, k_2\overline{\beta_P}/T_Y\right)$ as the parameter interval for β_{i3} ($i=H,T$), with $k_1=0.1$, $k_2=0.3$, T_Y representing the total years in the forecast, and $\overline{\beta_P}$ as the mean value of the transmission rate calibrated without the inclusion of this term. We assumed this reduction term only in the effect contact rate to avoid a model overfit, since few data was considered in the calibration process. Thus, for the transmission rates, we have

$$\beta_H(t)=\beta_{H1}+\beta_{H2}p(t)-\beta_{H3}t$$

and

$$\beta_T(t)=\beta_{T1}+\beta_{T2}p(t)-\beta_{T3}t$$

for the HIV/AIDS and TB models, respectively.

Parameter estimation

All datasets used in this article are publicly available. The demographic terms Λ and μ in the models (1) and (2) were estimated. The parameter μ was assumed as the inverse of the mean value of the Brazilian life expectancy between 2003 and 2030. The birth rate Λ was estimated to the total population from the model N approximates the Brazilian population size between 2003 and 2030.

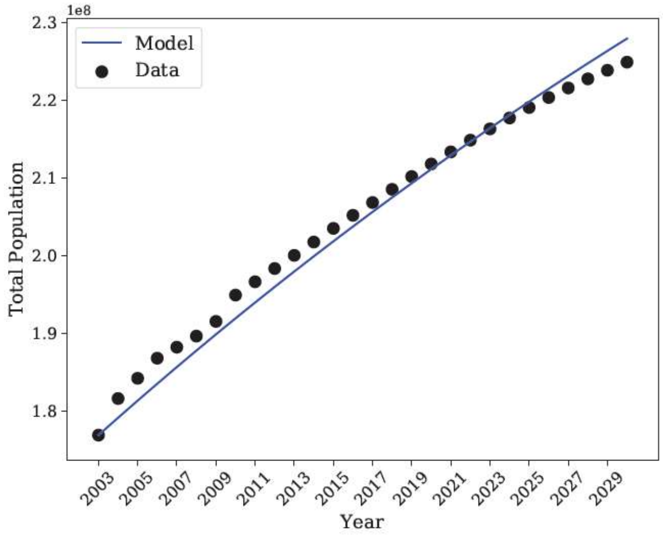

Figure S1: Brazilian total population fitting

Function representing the Brazilian total population fitting to the data and population projection until 2030 reported by Health Minister, in which the birth rate of the HIV/AIDS and TB models was estimated.

We calibrated the model (1) using the reported data of new AIDS cases and the annual AIDS-related deaths[17], represented by $\widehat{C}$ and $\widehat{D}$ respectively, between the years 2003 and 2019 (Supplementary Figure 20). Due to the COVID-19 pandemic, the process of reporting cases and deaths was compromised. Thus, we did not consider data for the year 2020. We included the following equations in the system (1),

$$\frac{dF_1}{dt} = \rho I + \alpha_2 T$$

$$\frac{dF_2}{dt} = \delta_1 A$$

where F_1 represents cumulative new cases and F_2 represents cumulative annual AIDS-related deaths. To compare annual data, we consider

$$C(i) = F_1(i) - F_1(i-1)$$

$$D(i) = F_2(i) - F_2(i-1)$$

which represents the new AIDS cases and annual deaths from the model (1).

Parameters presented in Table S1 were estimated by fitting the new AIDS cases and annual AIDS-related deaths. We used a genetic algorithm (GA) to fit the model to the data. A genetic algorithm is an optimization technique in which an initial population of "chromosomes" is generated and a proportion of them may survive to the next generation or be replaced by a new chromosome through the process of reproduction, crossover and mutation, depending on its fitness score[18]. The fitness score is given by the mean squared errors (RMSE)

$$E_C = \sqrt{\frac{1}{n}\sum_{i=1}^{n}\left[C(i) - \hat{C}(i)\right]^2}, E_D = \sqrt{\frac{1}{n}\sum_{i=1}^{n}\left[D(i) - \hat{D}(i)\right]^2}$$

In order to obtain the values of $C(i)$ and $D(i)$, we used the fourth-order Runge-Kutta method, where the initial population $I(0) = 2.6 \times 10^5$, $A(0) = 1.5 \times 10^5$, $C(0) = 38347$, $R(0) = 0$, $T(0) = 0.4\, I(0)$, $D(0) = 11{,}283$ and $S(0) = 1.77 \times 10^8 - I(0) - A(0) - T(0) - R(0)$. To perform the GA, we considered the package NSGA-II, which is a multiobjective genetic algorithm[19] for the Python programming language, with 1,500 generations and an initial population of 1.0×10^5 chromosomes to calibrate the model with the two datasets. To define the set of values for the parameters, we selected the smallest value of $E_C + E_D$, as this gave the smallest joint error for the fitness score.

For the TB model, the same methodology was applied:

$$\frac{dF_1}{dt} = \phi v P + \omega L$$

$$\frac{dF_2}{dt} = \mu_T I$$

Here, the parameters presented in Table S2 were estimated by fitting the TB incidence and annual deaths between 2003 and 2019[20] (Supplementary Figure 21). We applied the fourth-order Runge-Kutta method to solve the ODE system, assuming the

initial population $I(0)=78{,}615$, $L(0)=5\times10^{6}$, $P(0)=5\times10^{4}$, $C(0)=78{,}615$, $D(0)=4{,}987$ and $S(0)=1.77\times10^{8}-I(0)-L(0)-P(0)$ in order to define the TB parameter values fitted to the data.

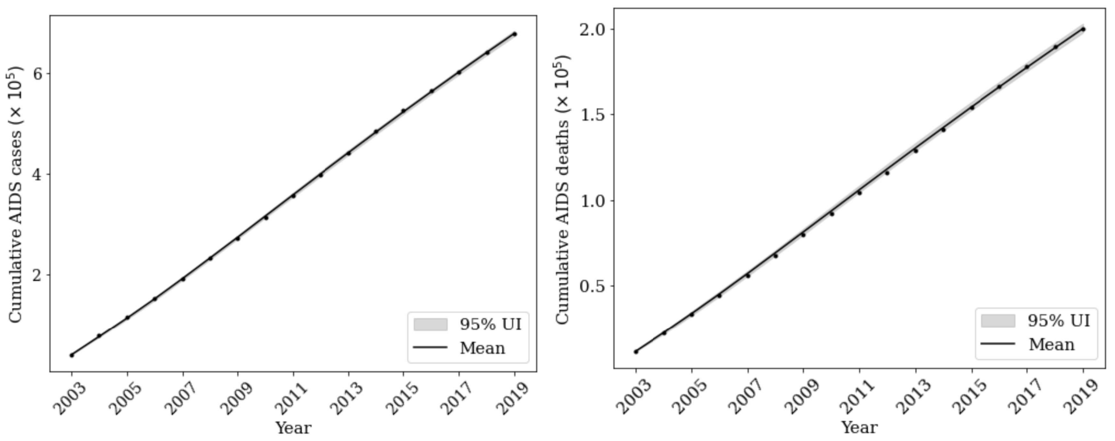

Figure S2: Cumulative AIDS cases and deaths fitted to the original data

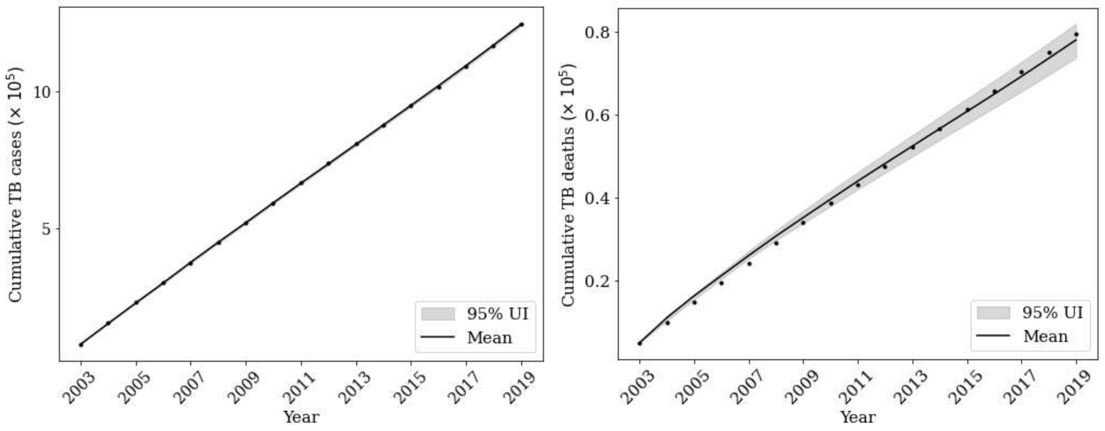

Figure S3: Cumulative TB cases and deaths fitted to the original data

HIV/AIDS model			
Parameter	Fitted Value	Parameter	Fitted Value
β_{H1}	0.7720191276399723	β_{H2}	1.7150001063069298
δ_{11}	0.07278722780683367	δ_{12}	0.0002086298599606487
α_{11}	0.25304785459146556	α_{12}	0.2514299908723004
α_{21}	0.05652379119844513	α_{22}	0.005508461518992459
ρ_1	0.09663797662616888	ρ_2	0.08445507759920495
γ_1	0.20000966124965328	γ_2	0.3226832108629463
β_{H3}	0.009639853084780438		

TB model			
Parameter	Fitted Value	Parameter	Fitted Value
β_{T1}	4.946975903274582	β_{T2}	5.604868403482449
v_1	3.965972468487727	v_2	3.343860671518547
ω_1	0.008321183714517056	ω_2	0.008695340464602526
μ_{T1}	0.07888362103526304	μ_{T2}	0.030851548732030783
τ_1	1.3356791773045626	τ_2	0.7267180369627797
β_{T3}	0.018062973755099455		

Table S3: Fitted parameters of the HIV/AIDS and TB models

Parameter values obtained by the multiobjective genetic algorithm in order to fit to the original TB and AIDS new cases and deaths.

Additionally, prediction intervals were estimated for these parameters in each model. The intervals were established using a non-parametric bootstrapping method with a Poisson distribution, considering 100 replicates of the original data of new cases and of AIDS-related and TB deaths as presented in Jorge et al. (2019)[21]. We assumed the original data as the mean parameter for the Poisson distribution. Figures S4 and S5 show the bootstrapping limits for each annual data according to the original data of AIDS and TB, respectively.

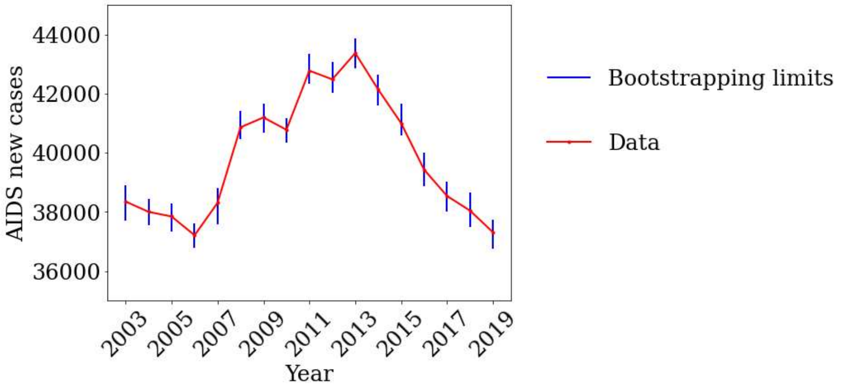

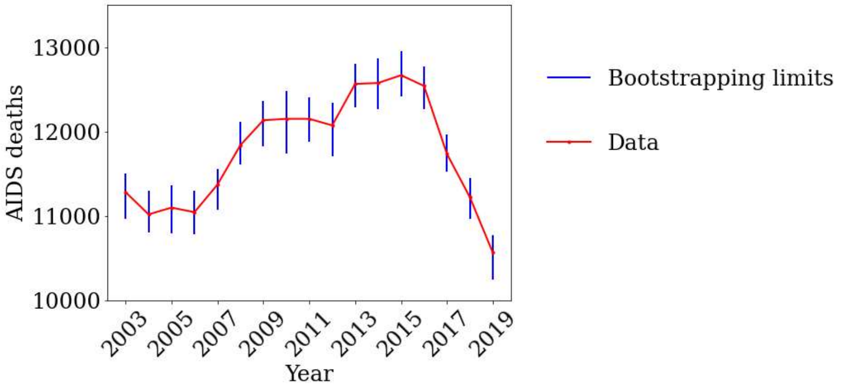

Figure S4: New AIDS cases and deaths from original data and limits obtained by the bootstrapping method

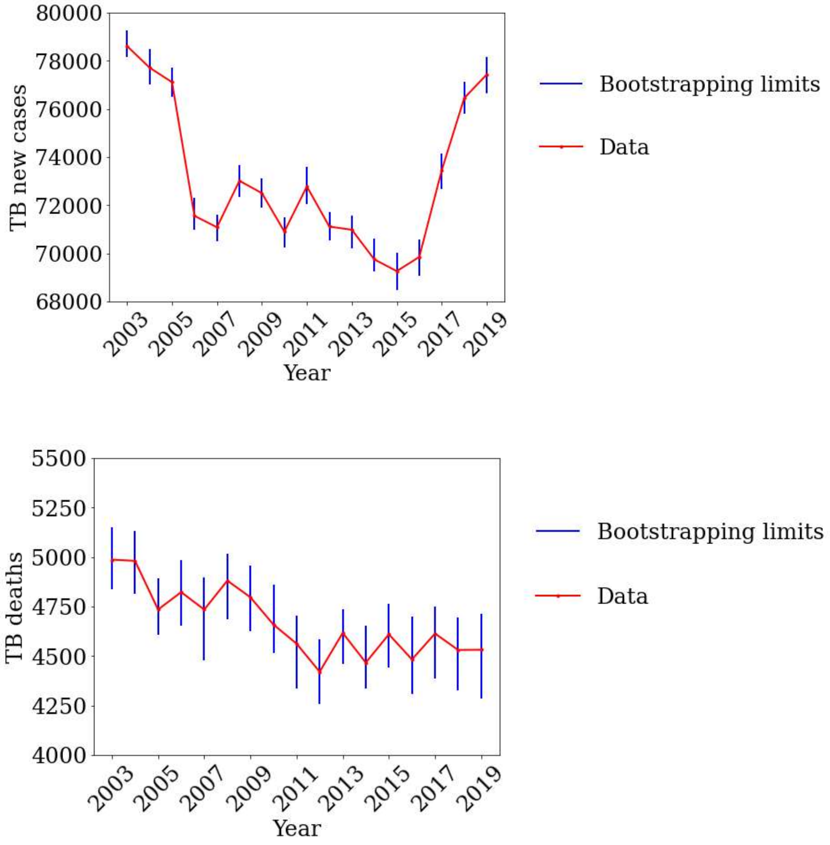

Figure S5: New TB cases and deaths from original data and limits obtained by the bootstrapping method

Forecast and scenarios of poverty rate

Data: We calculated poverty rates using three National Household Surveys (PNAD) – the regular PNAD, Continuous-PNAD, and PNAD-COVID for 2001-2011, 2012-2019, and 2020, respectively. These surveys are representative of the entire Brazilian population and are similar in structure, although they differ in terms of sample size and collection strategy. Data on benefits and eligibility criteria of the PBF were obtained from official documents published by the Ministry of Social Development.

Poverty Measurement Method: Poverty thresholds are used to determine the percentage of a population below specific levels of "economic wellbeing", measured by an individual's income. The eligibility criteria for participation in the PBF is to earn below the poverty line. In this study, we use the poverty lines stipulated as eligibility criteria for participation in the *Bolsa Família* program (BFP), which is the largest social protection in form of conditional cash transfer in Brazil and in the world. The poverty line values of the BFP are corrected for inflation by the Ministry of Social Development, although not on a yearly basis. These corrected values are used as the official national poverty lines in Brazil, and were used in this study to calculate the official poverty rate and vulnerable population officially considered eligible for social protection in Brazil. Therefore, these rates are more representative of the Brazilian poverty scenario and social protection compared to rates calculated using an international approach such as, for example, the World Bank's approach.

The poverty rate in year t was measured as the proportion of individuals that earn income below the poverty line, $Poor_t$, compared to the total Brazilian population, Pop_t, i.e., $Poor_t/Pop_t$. The poverty rate was obtained for the year 2010 by linear interpolation due to the lack of household surveys. We explored an international approach using the World Bank's Purchasing Power Parity (PPP). Although the poverty

time trend portrayed by the national and international approach (at $1.90 PPP) are similar (Figure S6), the *Bolsa Família* approach was a better representation of social protection in Brazil.

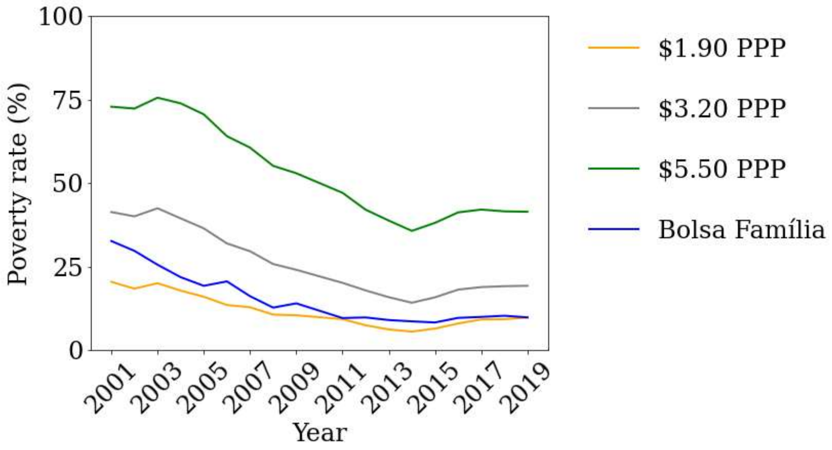

Figure S6: World Bank international poverty lines and *Bolsa Família* approach

Autoregressive approach: The poverty rates calculated from the year 2001 to 2020 are used to forecast values from 2021 to 2030 using Vector Autoregression (VAR) models represented as:

$$Y_t = \beta_0 + \beta_1 Y_{t-k} + trend + \varepsilon_t$$

where the dependent variable, Y_t, is the poverty rate for the year t. The regressors are the k lagged values of poverty rate, Y_{t-k}, and a trend, *trend*; ε_t is the error term that is assumed to be "white noise"[22].

Deterministic approach: In addition to the autoregressive approach, we forecasted the poverty rate using an exponential growth function. This deterministic approach is flexible enough to create a wide range of poverty scenarios and growth tendencies using specified parameters. The scenarios created in this study are grouped into two major categories – optimistic (where the poverty rate reduces) and pessimistic

(where the poverty rate increases). Optimistic scenarios are calculated using the following equation:

$$Pov_t = Pov_{t0} - C_i Pov_{t0}\left(1 - e^{-K_i t}\right)$$

whereas the pessimistic scenario is expressed as:

$$Pov_t = Pov_{t0} + C_i Pov_{t0}\left(1 - e^{-K_i t}\right).$$

Although the optimistic and pessimistic scenarios are simply mirrored equations, the behaviors of the projected trends depend on the values of the parameters detailed below

- t – time variable, whereby $t = \{2001, 2002, \ldots, 2030\}$ and $t0$ is the baseline or reference year;
- i – identifies growth. In this study, we had two options. $i=1$is high growth and $i=2$is low and steady growth. The time duration, t, is specified randomly and can be adjusted;
- Pov_t – is the calculated poverty rate at time or year t;
- Pov_{t0}– is the poverty rate at the baseline or reference year;
- C_i – represents the impact intensity the baseline poverty rate has on future rates for each growth option, and;
- K_i – represents the poverty rate increase for each growth option.

Using the exponential growth function, the parameters are specified to create four levels of social protection scenarios: two pessimistic scenarios (with poverty rate higher than the autoregressive approach) to represent an effect of no or almost none social protection, and two optimistic scenarios (with poverty rate smaller than the autoregressive approach) to represent the cases of a strong social protection.

		Scenarios	
		Optimistic	Pessimistic
Growth moment 1	K_1	1.5	1.5
	C_1	0.8	0.55
Growth moment 2	K_2	0.01	0.1
	C_2	1.5	0.53

Table S4: Parameter specification for the exponential growth function

Modelling poverty trends and projections with and without social protection: We used data from national household surveys to estimate the time series of poverty rate in Brazil since 2001. In 2020, we used individual-level data on income from nationwide surveys to estimate poverty with the introduction of the AE. We also estimated the poverty rate's trajectory if the AE had not been implemented by subtracting the AE benefits from household income. From 2020 to 2030, poverty rates were projected based on different simulation methods with five possible poverty scenarios: four deterministic scenarios developed by applying exponential decay formulas calibrated to the empirical data of 2019-2020, and one intermediate scenario derived by projections of autoregressive models applied to the previous poverty time series.

Figure S7 shows the poverty rate according to the level of social protection. The main text presents the three scenarios: no social protection, moderate social protection, and strong social protection.

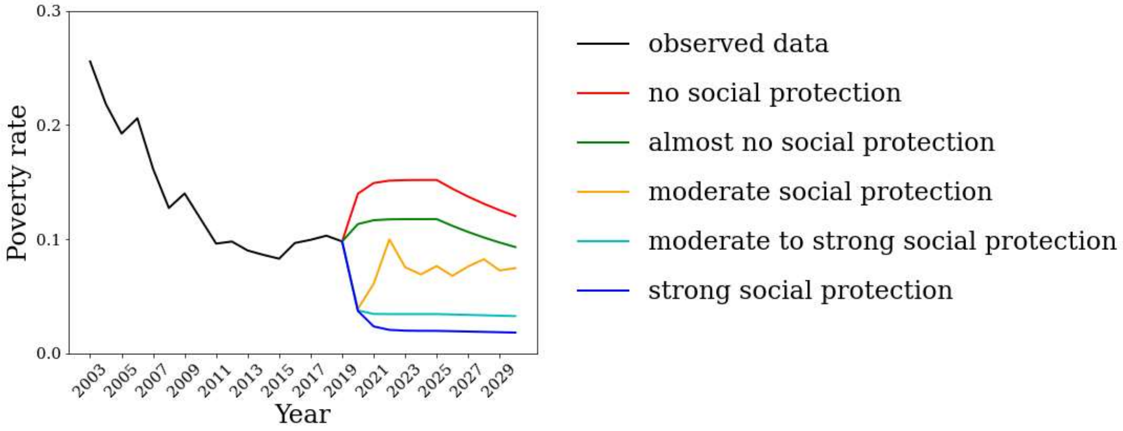

Figure S7: Poverty rate scenarios between 2003 and 2030 according to the level of social protection

Modelling HIV/AIDS and TB incidence and mortality rates

Here, we forecast the HIV/AIDS and TB incidence and mortality rates considering more two alternative levels of social protection between the no social protection scenario and the strong social protection scenario, totalling five possible scenarios (Figure S8).

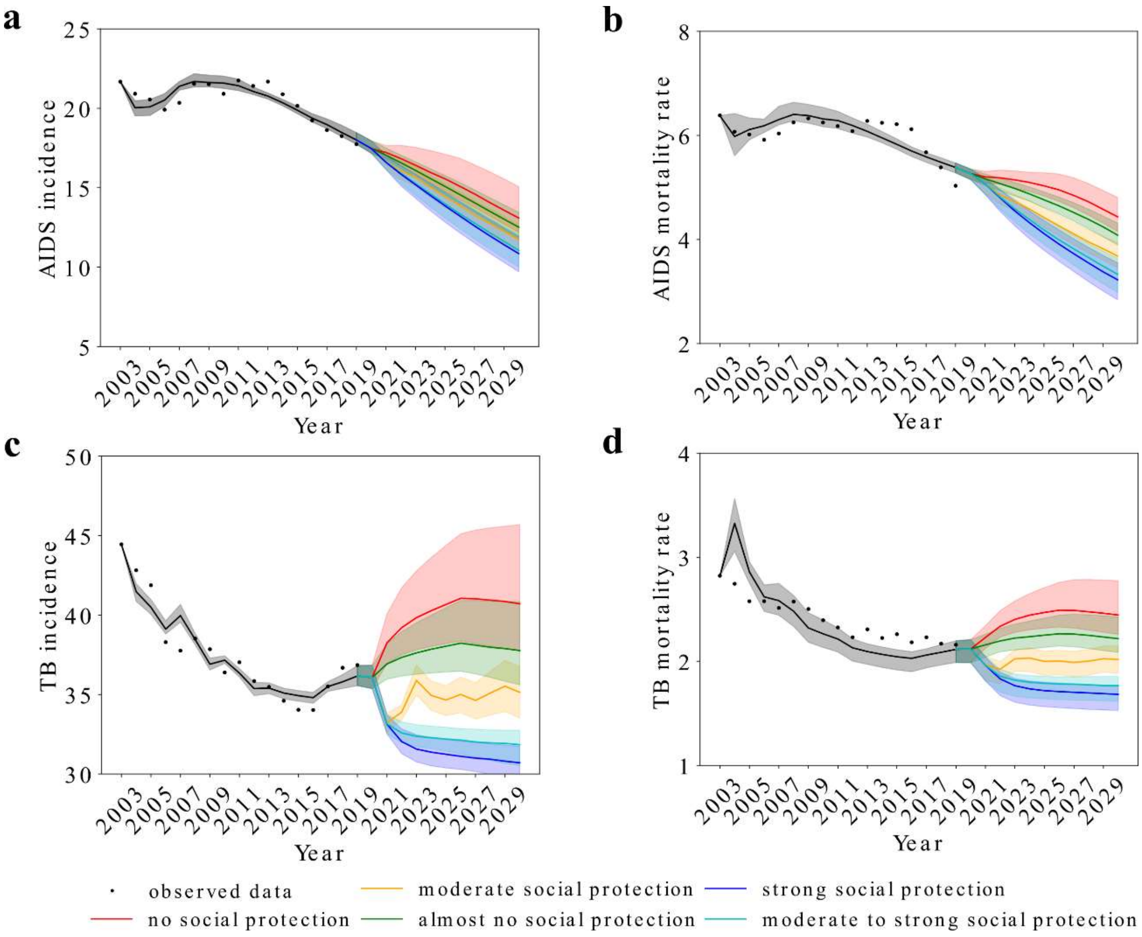

Figure S8: AIDS and Tuberculosis incidence and mortality

(**a**) HIV/AIDS incidence under different scenarios of poverty rates between 2003 and 2030, (**b**) HIV/AIDS mortality under different scenarios of poverty rates between 2003 and 2030, (**c**) Tuberculosis incidence under different scenarios of poverty rates between 2003 and 2030, and (**d**) Tuberculosis mortality under different scenarios of poverty rates between 2003 and 2030. Shaded bands represent 95% of simulated predictions according to the poverty rate.

Parameter sensitivity analysis

Here, a sensitivity analysis is performed to evaluate the effects of the mathematical model parameters in the dynamics of the all variables over time. By using a statistical variance-based method, described by Sobol (2001)[23], the sensitivity analysis of the HIV/AIDS and TB models is carry out.

We present the methodology for the TB model, but it can be extended for the HIV/AIDS model as well. In order to apply the sensitivity analysis for the TB model, we consider the following parameter vector

$$\Gamma = (\beta_T, \nu, \tau, \omega, \mu_T) \in R^8$$

(5)

assuming that its elements are uniformly distributed (U) in proper intervals presented in Table S2.

To apply the statistical variance-based method, sample values is generated for the input factors shown in Eq. (5) for TB model and Eq. (6) for HIV/AIDS model, respectively, by creating matrices A and B, each with size $N \times n$, where N is the number of samples, with $n=5$ for TB model and $n=6$ for HIV/AIDS model, respectively, is the number of parameters being analyzed, given by

$$A = \begin{pmatrix} \theta_1^{(A1)} & \theta_2^{(A1)} & \cdots & \theta_i^{(A1)} & \cdots & \theta_n^{(A1)} \\ \theta_1^{(A2)} & \theta_2^{(A2)} & \cdots & \theta_i^{(A2)} & \cdots & \theta_n^{(A2)} \\ \vdots & \vdots & \cdots & \vdots & \cdots & \vdots \\ \theta_1^{(AN)} & \theta_2^{(AN)} & \cdots & \theta_i^{(AN)} & \cdots & \theta_n^{(AN)} \end{pmatrix}$$

and

$$B = \begin{pmatrix} \theta_1^{(B1)} & \theta_2^{(B1)} & \cdots & \theta_i^{(B1)} & \cdots & \theta_n^{(B1)} \\ \theta_1^{(B2)} & \theta_2^{(B2)} & \cdots & \theta_i^{(B2)} & \cdots & \theta_n^{(B2)} \\ \vdots & \vdots & \cdots & \vdots & \cdots & \vdots \\ \theta_1^{(BN)} & \theta_2^{(BN)} & \cdots & \theta_i^{(BN)} & \cdots & \theta_n^{(BN)} \end{pmatrix}$$

We then create n matrices A_B^i, where column i comes from matrix B and all other $n-1$ columns come from matrix A:

$$A_B^i = \begin{pmatrix} \theta_1^{(A1)} & \theta_2^{(A1)} & \cdots & \theta_i^{(B1)} & \cdots & \theta_n^{(A1)} \\ \theta_1^{(A2)} & \theta_2^{(A2)} & \cdots & \theta_i^{(B2)} & \cdots & \theta_n^{(A2)} \\ \vdots & \vdots & \cdots & \vdots & \cdots & \vdots \\ \theta_1^{(AN)} & \theta_2^{(AN)} & \cdots & \theta_i^{(BN)} & \cdots & \theta_n^{(AN)} \end{pmatrix}$$

In the matrices A, B and A_B^i, each row represents a set of parameters to be used as an input for the model. Numerical simulations are performed, and the output of the sample matrices A, B and A_B^i are stored as the vectors

$$Y_A = \begin{pmatrix} Y\left(A^{(A1)}\right) \\ Y\left(A^{(A2)}\right) \\ \vdots \\ Y\left(A^{(AN)}\right) \end{pmatrix}, Y_B = \begin{pmatrix} Y\left(B^{(B1)}\right) \\ Y\left(B^{(B2)}\right) \\ \vdots \\ Y\left(B^{(BN)}\right) \end{pmatrix}, Y_{A_B^i}$$

where Y_A, Y_B and $Y_{A_B^i}$ are output vectors.

The final step involves the calculation of the sensitivity indices, using the samples generated during the sampling scheme. We computed the total effect indices, given by

$$S_{T_i} = 1 - \frac{Y_A \cdot Y_B - f^2}{Y_A \cdot Y_A - f^2}$$

where f is defined as

$$f := \frac{1}{N} \sum_{j=1}^{N} Y_A^{(j)}$$

This index indicates the contribution of the parameter to the output of the model. The importance of each parameter i is proportional to the value of S_{T_i}, meaning that higher S_{T_i} leads to a higher contribution to the model output[24]. Parameters with higher S_T need a more carefully calibration, as small error during the calibration can lead to larger errors to the predictions generated by the model. The total effect takes into account higher-order interactions among model variables; thus, correlation between variables can also be identified using this method.

In Figures S9 to S16 the result of the sensitivity analysis over time for the TB model is presented. The numerical simulations were performed using ODEsensitivity[25] library from R language. The experiment was conducted generating $N = 100{,}000$ parameter combinations, totaling 500,000 simulations of the model, and the result shows the evolution of the parameters according to S, P, I, and L compartments. The results for the sensitivity analysis of the TB model shows that the rate of progression from primary infection, ν, is the most important parameter over all system followed by the TB transmission rate β_T.

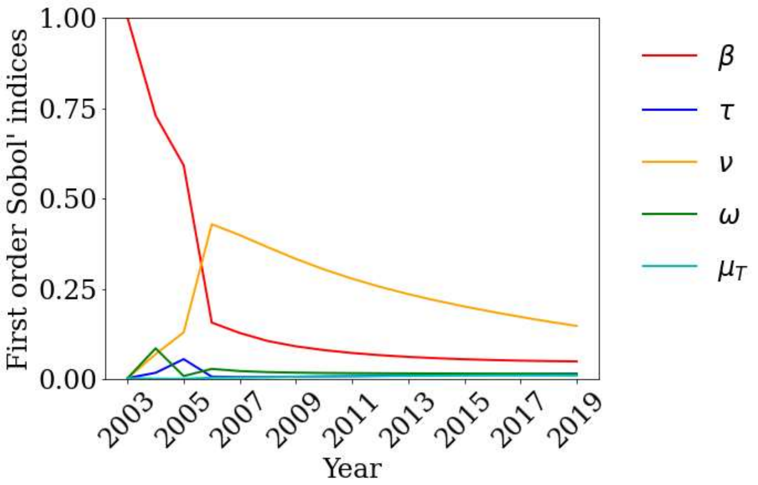

Figure S9: First order Sobol' indices for the S compartment of the TB model

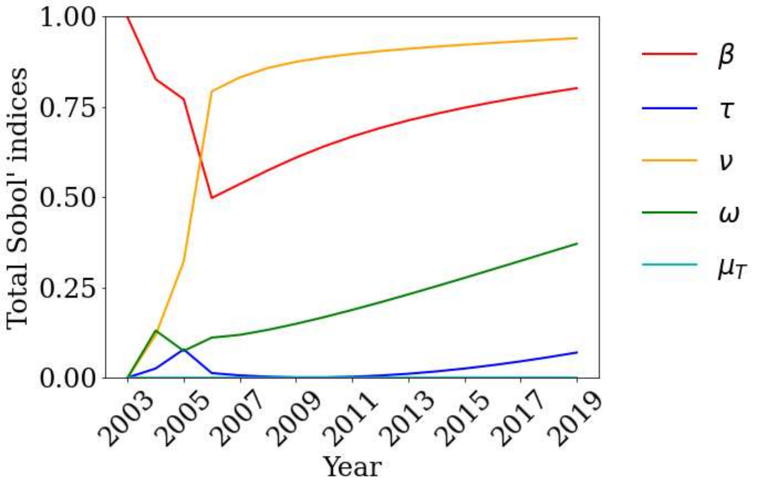

Figure S10: Total Sobol' indices for the S compartment of the TB model

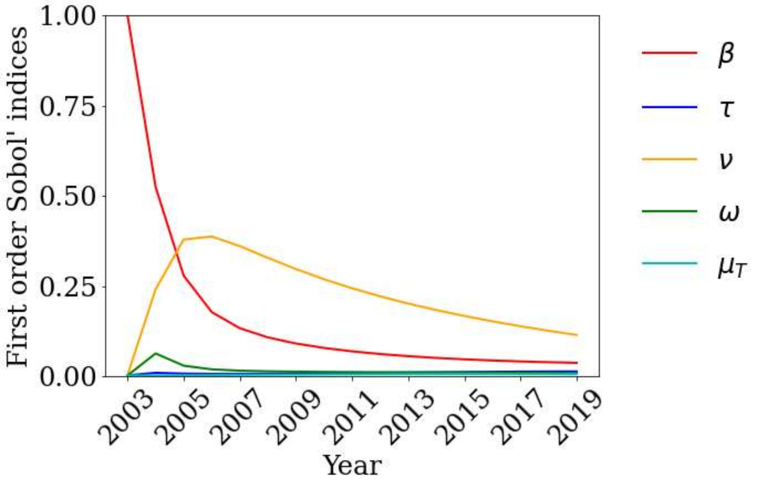

Figure S11: First order Sobol' indices for the P compartment of the TB model

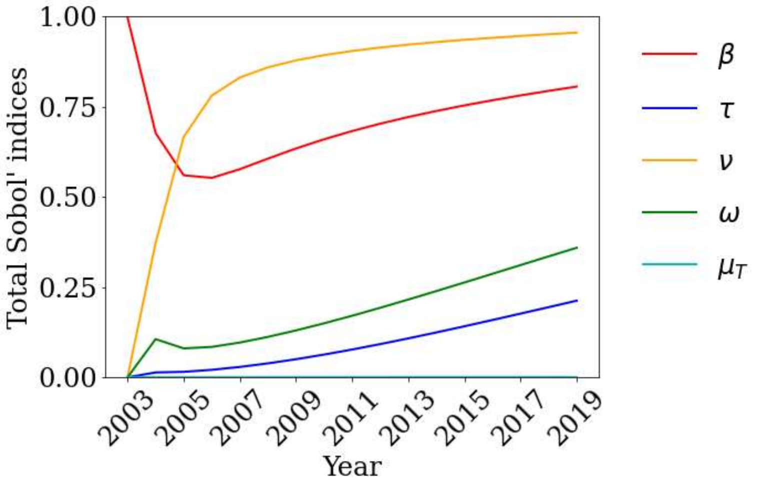

Figure S12: Total Sobol' indices for the P compartment of the TB model

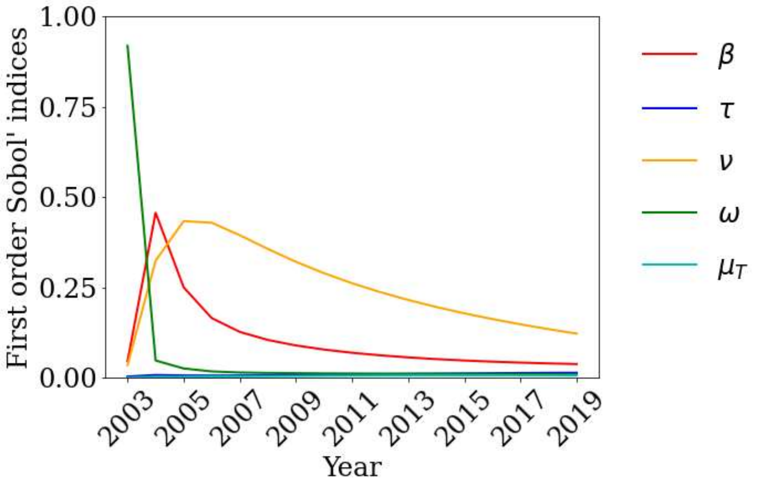

Figure S13: First order Sobol' indices for the L compartment of the TB model

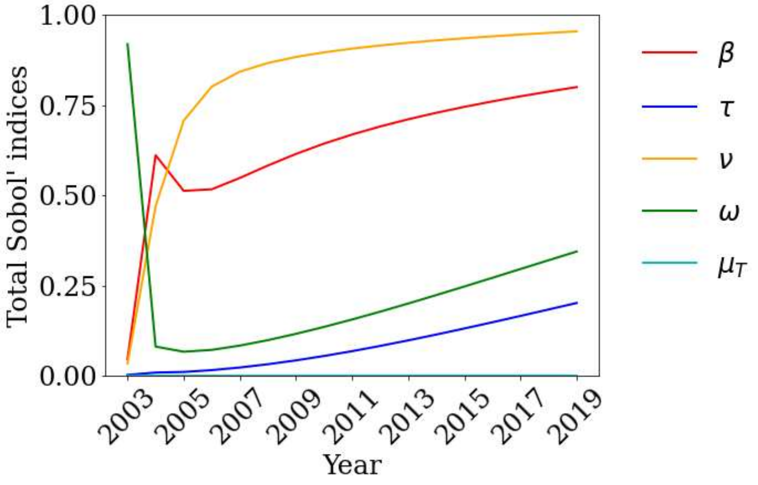

Figure S14: Total Sobol' indices for the L compartment of the TB model

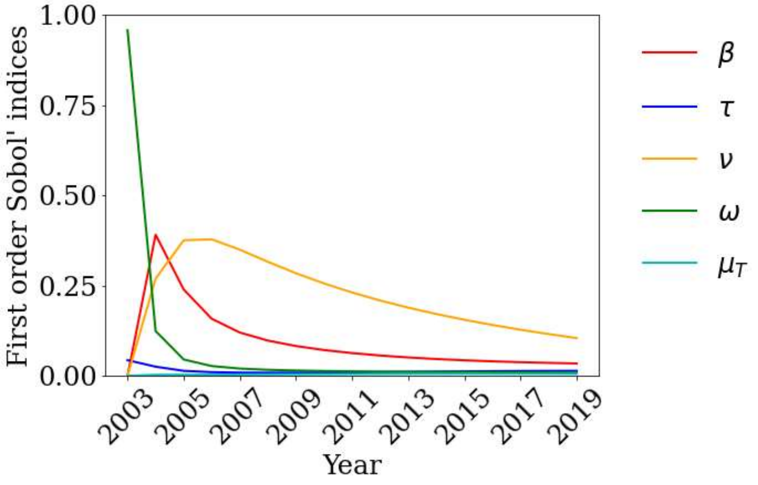

Figure S15: First order Sobol' indices for the I compartment of the TB model

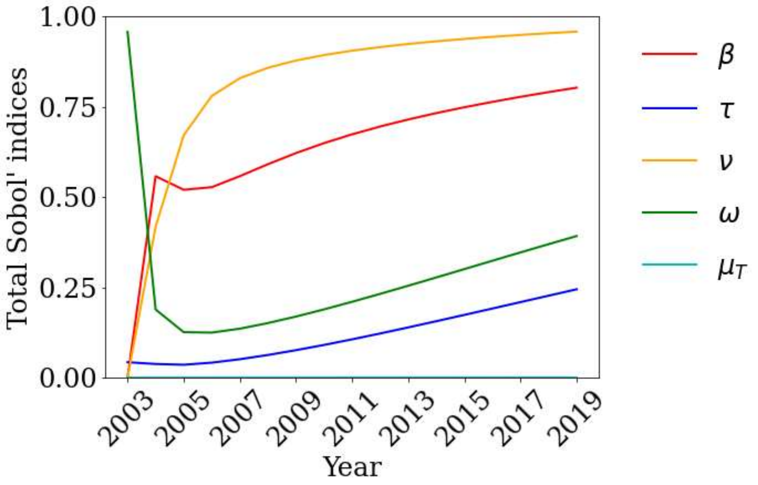

Figure S16: Total Sobol' indices for the I compartment of the TB model

For the HIV/AIDS model we consider the parameter vector

$$\Gamma := (\beta_H, \rho, \alpha_1, \alpha_2, \delta_1, \gamma) \in R^{10} \tag{6}$$

assuming that its elements are uniformly distributed (U) in proper intervals presented in Tables 1. The experiment was conducted generating $N = 100{,}000$ parameter combinations, totaling 600,000 simulations of the HIV/AIDS model. Figures S17 to S26 show the result of the sensitivity analysis for the HIV/AIDS model. Here, the parameter that most influences the variation of the variable over time is the HIV transmission rate β_H.

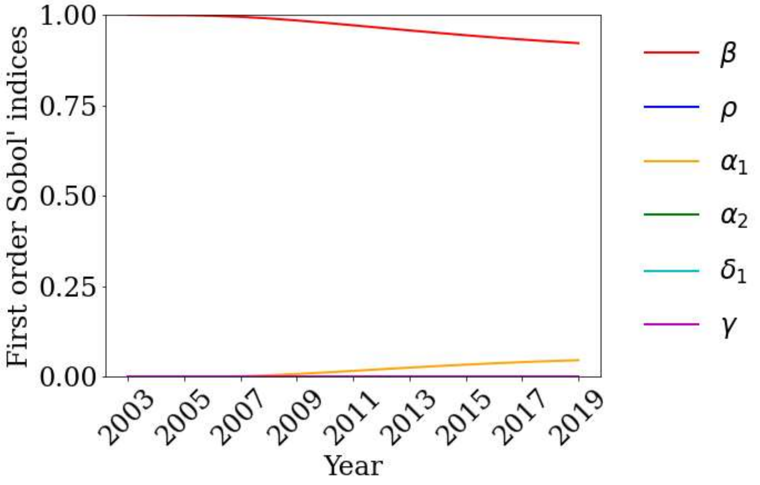

Figure S17: First order Sobol' indices for the S compartment of the HIV/AIDS model

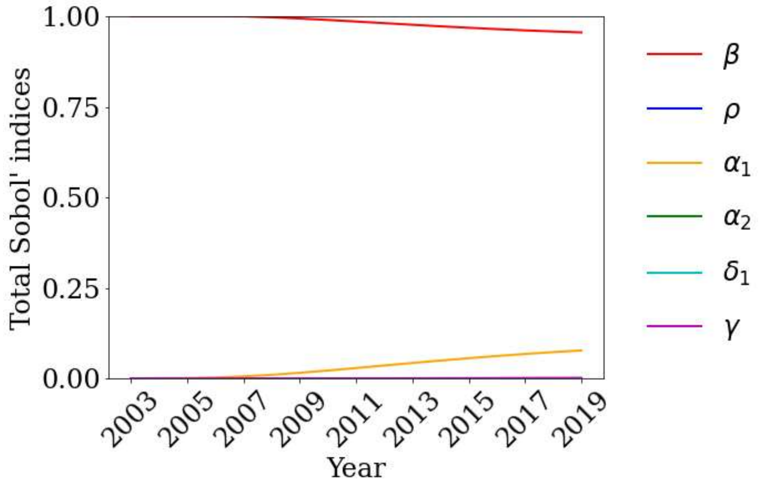

Figure S18: Total Sobol' indices for the S compartment of the HIV/AIDS model

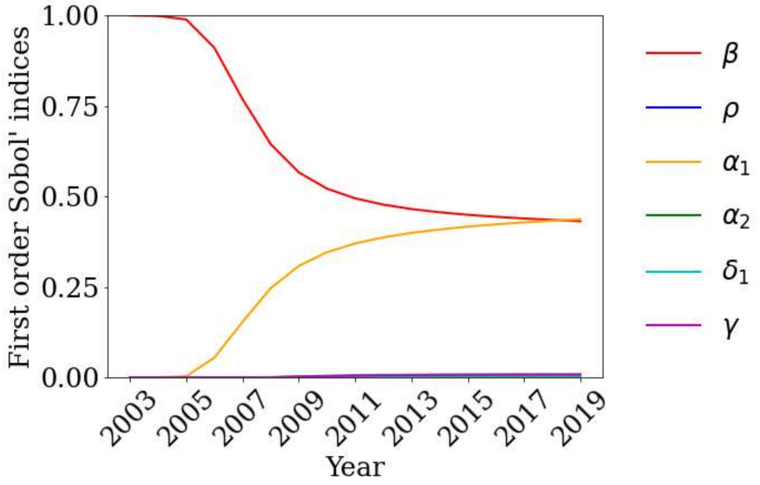

Figure S19: First order Sobol' indices for the I compartment of the HIV/AIDS model

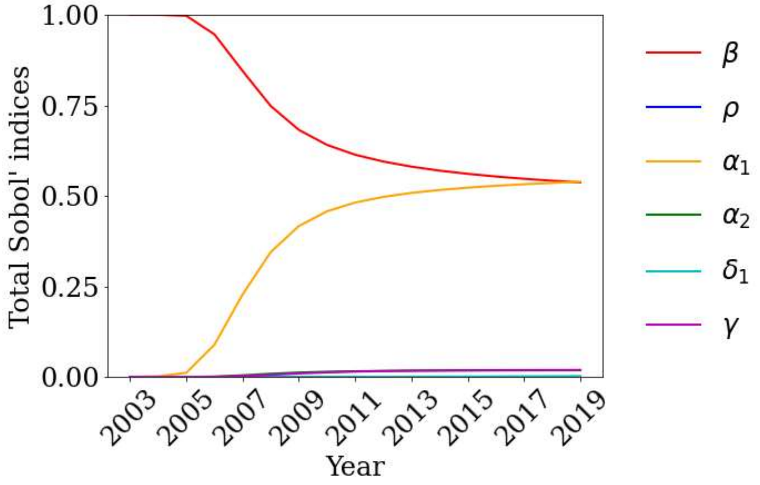

Figure S20: Total Sobol' indices for the I compartment of the HIV/AIDS model

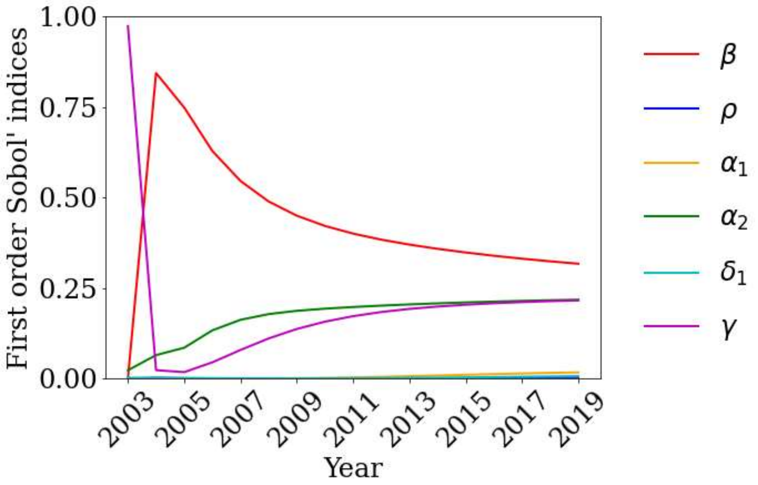

Figure S21: First order Sobol' indices for the A compartment of the HIV/AIDS model

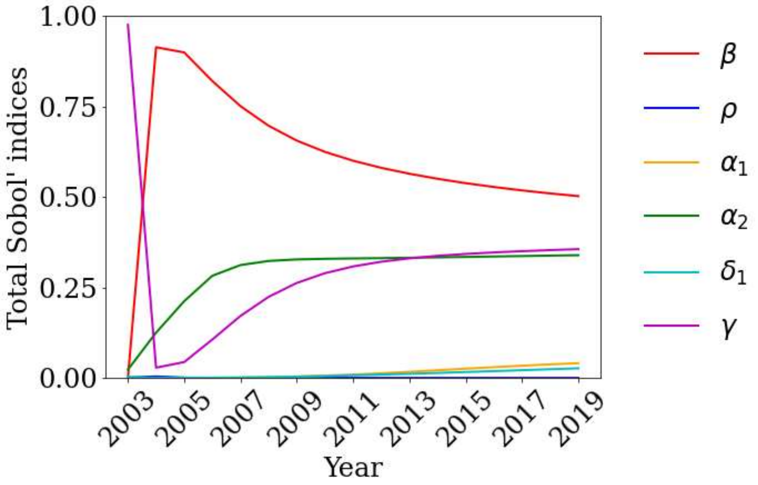

Figure S22: Total Sobol' indices for the A compartment of the HIV/AIDS model

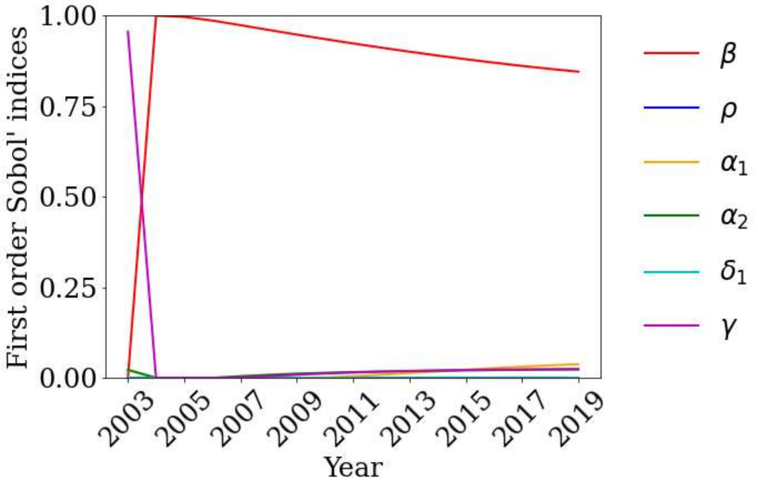

Figure S23: First order Sobol' indices for the T compartment of the HIV/AIDS model

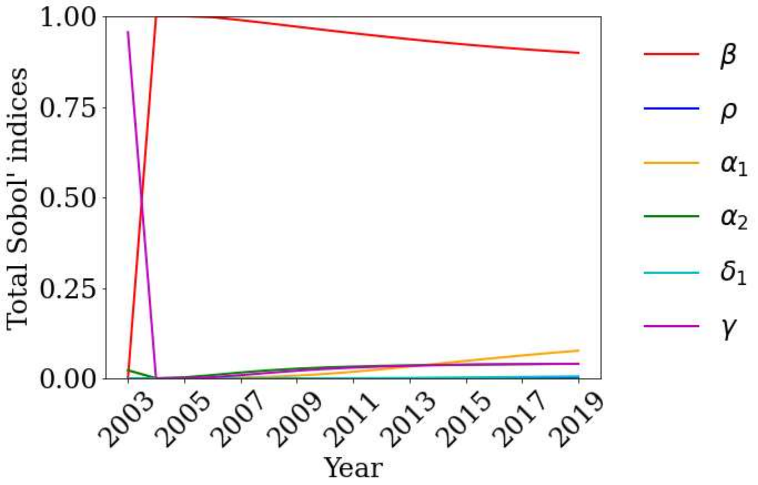

Figure S24: Total Sobol' indices for the T compartment of the HIV/AIDS model

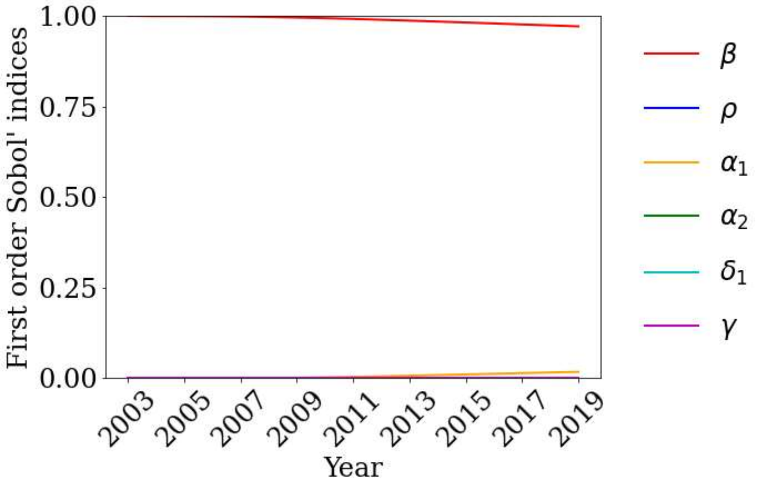

Figure S25: First order Sobol' indices for the R compartment of the HIV/AIDS model

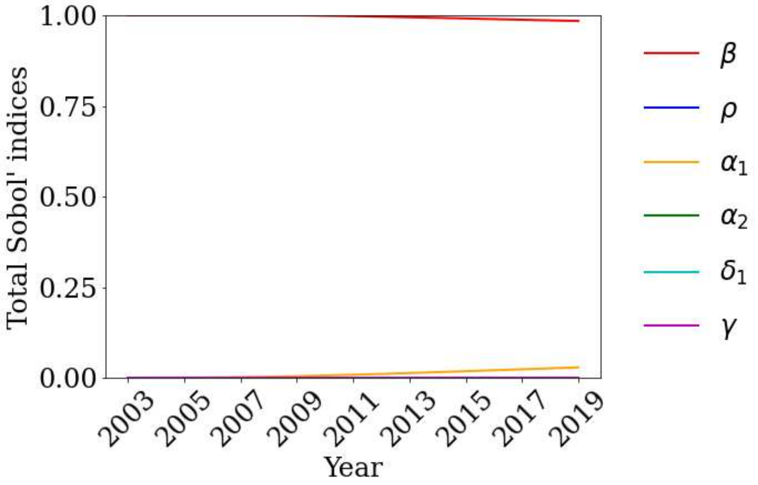

Figure S26: Total Sobol' indices for the R compartment of the HIV/AIDS model

Theoretical Model of the effects of Poverty on HIV/AIDS and Tuberculosis

Poverty is the most impactful social determinant of health (SDH) in the majority of poverty-related diseases.[26] The main reason is that it is one of the SDH positioned more upstream in the causal pathway of the social determination of diseases, and it is the one that is influencing the largest number of subsequent proximal risk factors.[27] Poverty status can also be considered an indicator, or proxy, of socioeconomic vulnerability and low socioeconomic position, representing in the models more than monetary poverty.[28]

In Tuberculosis, as shown in the theoretical framework of Figure S27, poverty can act as upstream factor of several proximal risk factors of different stages of the disease evolution: crowding and poor ventilation due to inadequate household infrastructure are associated with the exposure to TB, while smoking, indoor air pollution, malnutrition, lung and other diseases can impair the lung health and host defences of the individuals and promote the development of active TB, also acting in the evolution of active TB to an eventual cure, abandonment of treatment and/or death.[29–31]

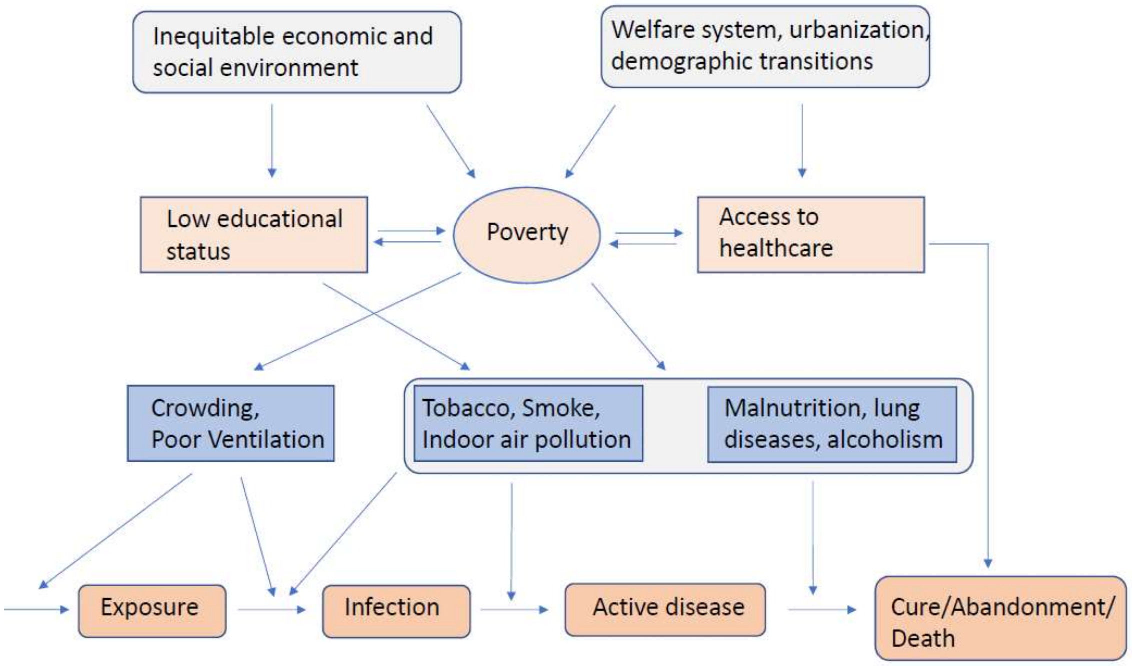

Figure 27S: Theoretical framework of the effects of poverty on different stages of the TB disease.

The access to healthcare, also associated with economic barriers and poverty, strongly influence the evolution of the active disease to its final outcomes.

In HIV/AIDS, as shown in the theoretical framework of Figure S28, poverty can act as upstream factor of proximal risk factors of different stages of the disease evolution:[32–34] risky sexual behaviours are associated with the exposure to HIV, and are influenced by poverty (through commercial sex work or transactional sex, especially of socioeconomically vulnerable women), low educational status (also determined by poverty), and access to condoms or other protective devices or treatment (such as Pre-exposure prophylaxis – PrEP). Improvements of nutritional and health status,[35] also associated to poverty, can delay or inhibit the conversion from HIV infection to AIDS, as well as the progression from AIDS to viral load suppression, or abandonment and death.

Also access to healthcare, determined by economic barriers, can influence these two last evolutions of the HIV/AIDS disease.

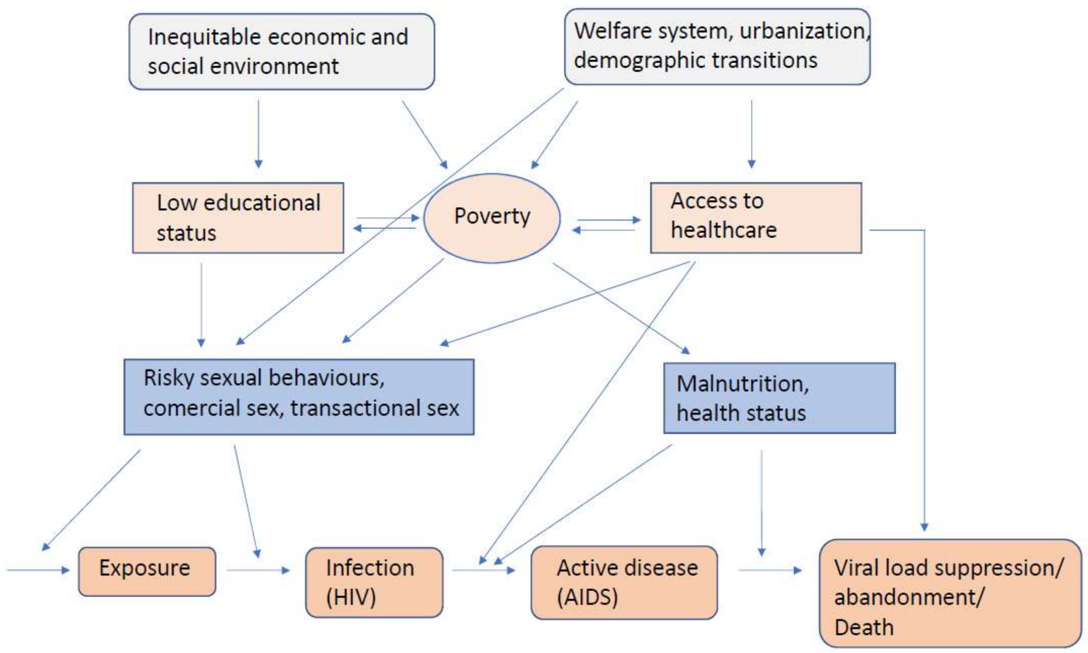

Figure 28S: Theoretical framework of the effects of poverty on different stages of the HIV/AIDS disease.

Among the upstream factors that more influence the HIV/AIDS and TB evolution, poverty is the most volatile in times of economic crises and the one that can be more quickly improved (through cash transfers for example), while educational status and healthcare infrastructure usually demand expensive, long-term and more burdensome interventions.

References

1 Huo HF, Chen R, Wang XY. Modelling and stability of HIV/AIDS epidemic model with treatment. *Applied Mathematical Modelling* 2016; **40**: 6550–9.

2 IBGE | Projeção da população. https://www.ibge.gov.br/apps/populacao/projecao/index.html (accessed May 18, 2022).

3 HIV/AIDS. https://www.who.int/news-room/questions-and-answers/item/hiv-aids (accessed May 18, 2022).

4 Ali JH, Yirtaw TG. Time to viral load suppression and its associated factors in cohort of patients taking antiretroviral treatment in East Shewa zone, Oromiya, Ethiopia, 2018. *BMC Infectious Diseases* 2019; **19**: 1–6.

5 Leng X, Liang S, Ma Y, *et al.* HIV virological failure and drug resistance among injecting drug users receiving first-line ART in China. *BMJ Open* 2014; **4**: e005886.

6 Jobanputra K, Parker LA, Azih C, *et al.* Factors Associated with Virological Failure and Suppression after Enhanced Adherence Counselling, in Children, Adolescents and Adults on Antiretroviral Therapy for HIV in Swaziland. *PLOS ONE* 2015; **10**: e0116144.

7 Hassan AS, Nabwera HM, Mwaringa SM, *et al.* HIV-1 virologic failure and acquired drug resistance among first-line antiretroviral experienced adults at a rural HIV clinic in coastal Kenya: A cross-sectional study. *AIDS Research and Therapy* 2014; **11**: 1–12.

8 Rupérez M, Pou C, Maculuve S, *et al.* Determinants of virological failure and antiretroviral drug resistance in Mozambique. *Journal of Antimicrobial Chemotherapy* 2015; **70**: 2639–47.

9 J T, JR G, M M, *et al.* Time from HIV seroconversion to death: a collaborative analysis of eight studies in six low and middle-income countries before highly active antiretroviral therapy. *AIDS (London, England)* 2007; **21 Suppl 6**. DOI:10.1097/01.AIDS.0000299411.75269.E8.

10 CDC. Evidence of HIV Treatment and Viral Suppression in Preventing the Sexual Transmission of HIV. 2020.

11 Gomes MGM, Oliveira JF, Bertolde A, *et al.* Introducing risk inequality metrics in tuberculosis policy development. *Nature Communications 2019 10:1* 2019; **10**: 1–12.

12 Haley CA. Treatment of Latent Tuberculosis Infection. *Microbiology Spectrum* 2017; **5**. DOI:10.1128/MICROBIOLSPEC.TNMI7-0039-2016.

13 Tang P, Johnston J. Treatment of Latent Tuberculosis Infection. *Current Treatment Options in Infectious Diseases 2017 9:4* 2017; **9**: 371–9.

14 Garziera G, Morsch ALB, Otesbelgue F, *et al.* Latent tuberculosis infection and tuberculosis in patients with rheumatic diseases treated with anti-tumor necrosis factor agents. *Clinical Rheumatology 2017 36:8* 2017; **36**: 1891–6.

15 Mack U, Migliori GB, Sester M, *et al.* LTBI: latent tuberculosis infection or lasting immune responses to M. tuberculosis? A TBNET consensus statement. *European Respiratory Journal* 2009; **33**: 956–73.

16 Global tuberculosis report 2018. https://apps.who.int/iris/handle/10665/274453 (accessed May 18, 2022).

17 Boletim Epidemiológico de Turbeculose 2020 | Departamento de Doenças de Condições Crônicas e Infecções Sexualmente Transmissíveis. http://www.aids.gov.br/pt-br/pub/2020/boletim-epidemiologico-de-turbeculose-2020 (accessed May 18, 2022).

18 Whitley D. A genetic algorithm tutorial. *Statistics and Computing 1994 4:2* 1994; **4**: 65–85.

19 Deb K, Pratap A, Agarwal S, Meyarivan T. A fast and elitist multiobjective genetic algorithm: NSGA-II. *IEEE Transactions on Evolutionary Computation* 2002; **6**: 182–97.

20 DATASUS – Ministério da Saúde. https://datasus.saude.gov.br/ (accessed May 18, 2022).

21 Jorge DCP, Rodrigues MS, Silva MS, *et al.* Assessing the nationwide impact of COVID-19 mitigation policies on the transmission rate of SARS-CoV-2 in Brazil. *Epidemics* 2021; **35**: 100465.

22 Lütkepohl H. New introduction to multiple time series analysis. *New introduction to Multiple Time Series Analysis* 2005; : 1–764.

23 Sobol IM. Global sensitivity indices for nonlinear mathematical models and their Monte Carlo estimates. *Mathematics and Computers in Simulation* 2001; **55**: 271–80.

24 Saltelli A, Ratto M, Andres T, *et al.* Global sensitivity analysis: The primer. *Global Sensitivity Analysis: The Primer* 2008; : 1–292.

25 Weber F, Theers S, Surmann D, Ligges U, Weihs C. Eldorado: Sensitivity Analysis of Ordinary Differential Equation Models. https://eldorado.tu-dortmund.de/handle/2003/36875 (accessed June 10, 2022).

26 Health WC on SD of, Organization WH. Closing the Gap in a Generation: Health Equity Through Action on the Social Determinants of Health : Commission on Social Determinants of Health Final Report. World Health Organization, 2008.

27 Chokshi DA. Income, Poverty, and Health Inequality. *JAMA* 2018; **319**: 1312–3.

28 Radosavljevic S, Haider LJ, Lade SJ, Schlüter M. Implications of poverty traps across levels. *World Development* 2021; **144**: 105437.

29 Carter DJ, Glaziou P, Lönnroth K, *et al.* The impact of social protection and poverty elimination on global tuberculosis incidence: a statistical modelling analysis of Sustainable Development Goal 1. *The Lancet Global Health* 2018; **6**: e514–22.

30 Carter DJ, Daniel R, Torrens AW, *et al.* The impact of a cash transfer programme on tuberculosis treatment success rate: A quasi-experimental study in Brazil. *BMJ Global Health* 2019; **4**. DOI:10.1136/bmjgh-2018-001029.

31 Hargreaves JR, Boccia D, Evans CA, Adato M, Petticrew M, Porter JDH. The Social Determinants of Tuberculosis: From Evidence to Action. *Am J Public Health* 2011; **101**: 654–62.

32 Dean HD, Fenton KA. Addressing social determinants of health in the prevention and control of HIV/AIDS, viral hepatitis, sexually transmitted infections, and tuberculosis. *Public Health Rep* 2010; **125 Suppl 4**: 1–5.

33 Morais GA de S, Magno L, Silva AF, *et al.* Effect of a conditional cash transfer programme on AIDS incidence, hospitalisations, and mortality in Brazil: a longitudinal ecological study. *The Lancet HIV* 2022; **9**: e690–9.

34 Stoner MCD, Kilburn K, Godfrey-Faussett P, Ghys P, Pettifor AE. Cash transfers for HIV prevention: A systematic review. *PLOS Medicine* 2021; **18**: e1003866.

35 Forrester JE, Sztam KA. Micronutrients in HIV/AIDS: is there evidence to change the WHO 2003 recommendations?1234. *Am J Clin Nutr* 2011; **94**: 1683S-1689S.
